# Comprehensive investigation of a novel differentially expressed lncRNA expression profile signature to assess the survival of patients with colorectal adenocarcinoma

**DOI:** 10.18632/oncotarget.15161

**Published:** 2017-02-06

**Authors:** Jiang-Hui Zeng, Liang Liang, Rong-Quan He, Rui-Xue Tang, Xiao-Yong Cai, Jun-Qiang Chen, Dian-Zhong Luo, Gang Chen

**Affiliations:** ^1^ Department of Pathology, First Affiliated Hospital of Guangxi Medical University, Nanning, Guangxi Zhuang Autonomous Region, P. R. China; ^2^ Department of General Surgery, First Affiliated Hospital of Guangxi Medical University (West Branch), Nanning, Guangxi Zhuang Autonomous Region, P. R. China; ^3^ Department of Medical Oncology, First Affiliated Hospital of Guangxi Medical University, Nanning, Guangxi Zhuang Autonomous Region, P. R. China; ^4^ Department of Gastrointestinal Surgery, First Affiliated Hospital of Guangxi Medical University, Nanning, Guangxi Zhuang Autonomous Region, P. R. China

**Keywords:** lncRNA, COAD, READ, prognostic biomarker, survival

## Abstract

Growing evidence has shown that long non-coding RNAs (lncRNAs) can serve as prospective markers for survival in patients with colorectal adenocarcinoma. However, most studies have explored a limited number of lncRNAs in a small number of cases. The objective of this study is to identify a panel of lncRNA signature that could evaluate the prognosis in colorectal adenocarcinoma based on the data from The Cancer Genome Atlas (TCGA). Altogether, 371 colon adenocarcinoma (COAD) patients with complete clinical data were included in our study as the test cohort. A total of 578 differentially expressed lncRNAs (DELs) were observed, among which 20 lncRNAs closely related to overall survival (OS) in COAD patients were identified using a Cox proportional regression model. A risk score formula was developed to assess the prognostic value of the lncRNA signature in COAD with four lncRNAs (LINC01555, RP11-610P16.1, RP11-108K3.1 and LINC01207), which were identified to possess the most remarkable correlation with OS in COAD patients. COAD patients with a high-risk score had poorer OS than those with a low-risk score. The multivariate Cox regression analyses confirmed that the four-lncRNA signature could function as an independent prognostic indicator for COAD patients, which was largely mirrored in the validating cohort with rectal adenocarcinoma (READ) containing 158 cases. In addition, the correlative genes of LINC01555 and LINC01207 were enriched in the cAMP signaling and mucin type O-Glycan biosynthesis pathways. With further validation in the future, our study indicates that the four-lncRNA signature could serve as an independent biomarker for survival of colorectal adenocarcinoma.

## INTRODUCTION

Colon adenocarcinoma (COAD) is one of the most frequently diagnosed cancers and a top cause of cancer death globally. Approximately 1.2 million new cases are diagnosed, causing 0.6 million deaths per year all over the world. Cancer metastasis remains the key cause of COAD death [1–6]. The five-year overall survival (OS) rate of patients with primary COAD can be up to 80–90%, but it is reduced to 5–10% in patients with metastatic tumor [7–12]. Rectal adenocarcinoma (READ), which shares similar molecular mechanism with COAD, has a comparable high incidence and poor prognosis [1–4, 6–8, 10]. Therefore, the assessment of prognostic factors is pivotal for the management of unresectable colorectal cancer patients. Several underlying mechanisms have been described in the past decades, including multiple molecular alterations, which confer the tumorigenesis and progression of colorectal cancer [13–18]. However, the exact underlying molecular markers for survival assessment of colorectal cancer remain largely unknown.

Long non-coding RNAs (lncRNAs) are a class of non-protein coding RNAs of more than 200 nucleotides, which are broadly distributed in the genome and can modulate gene expression [19–22]. Recent accumulating evidence has demonstrated that lncRNA expression profiles are frequently changed in tumors compared to that of the adjacent non-tumorous tissues in numerous cancers [23–27]. The altered lncRNA expression profile has been proposed to correlate with the progression and survival in patients with various cancers, including colorectal cancer, which reveals the potential of lncRNAs to act as cancer biomarkers [28–35]. However, most of the previous studies explored a limited number of lncRNAs in a small number of cases [36–39].

Previous studies have verified that a lncRNA expression signature could be obtained from the database of The Cancer Genome Atlas (TCGA), which offers a platform for researchers to download and assess free public datasets [40–45]. Towards this, in the current study, the TCGA database was first applied to gather lncRNA gene expression profiles in COAD. By performing a comprehensive lncRNA expression profile assessment, we identified a lncRNA signature in COAD with four lncRNAs (LINC01555, RP11-610P16.1, RP11-108K3.1, and LINC01207), as a new candidate indicator with the potential to predict the OS in COAD patients. Furthermore, the prognostic value of this four-lncRNA-signature was validated in a READ cohort (Supplementary Figure 1).

## RESULTS

### Differentially expressed lncRNAs (DELs)

**Figure 1:**
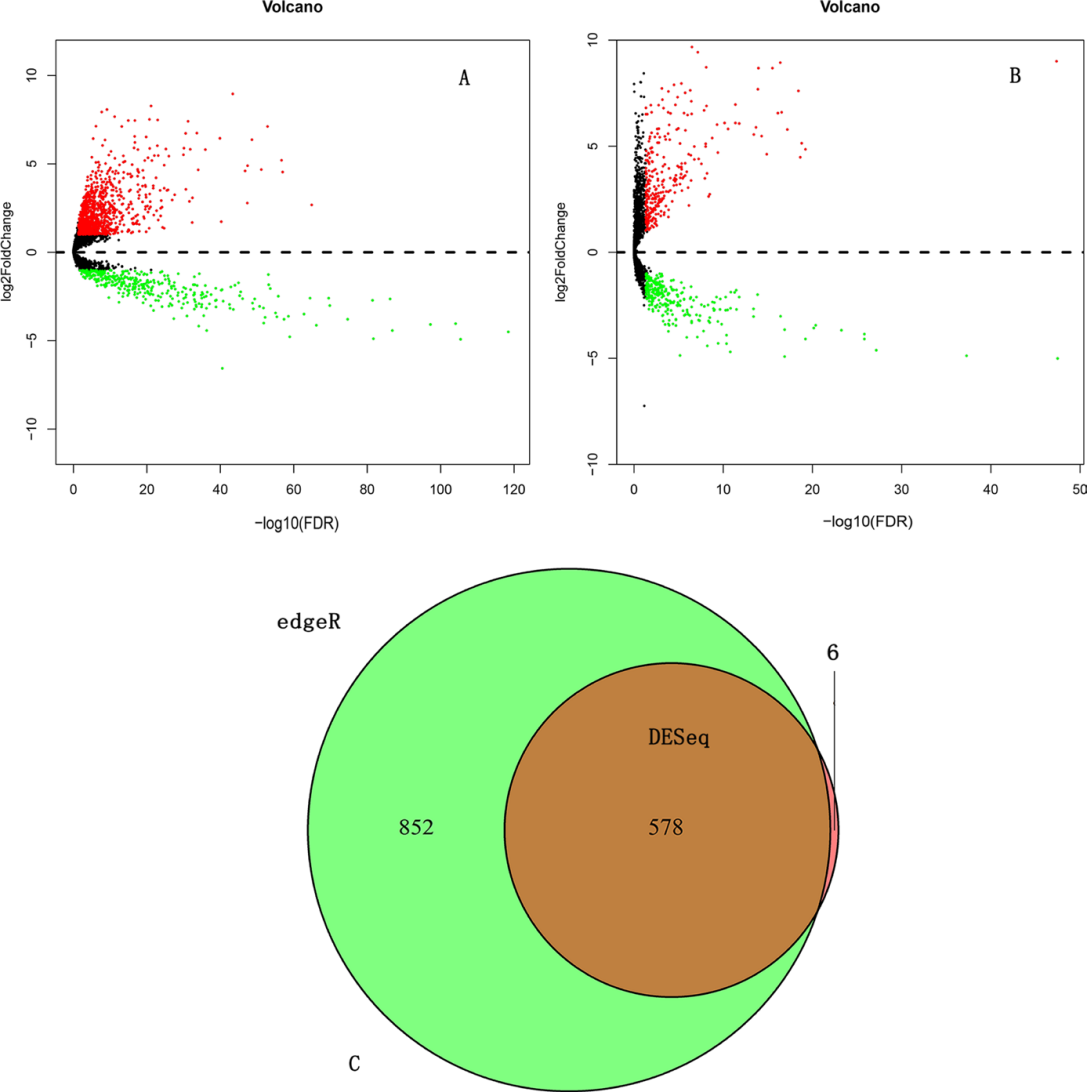
Differentially expressed lncRNAs (DELs) analysis (**A**) DELs identified using the edgeR package; (**B**) DELs identified using the DESeq package; (**C**) Overlapping DELs.

**Figure 2:**
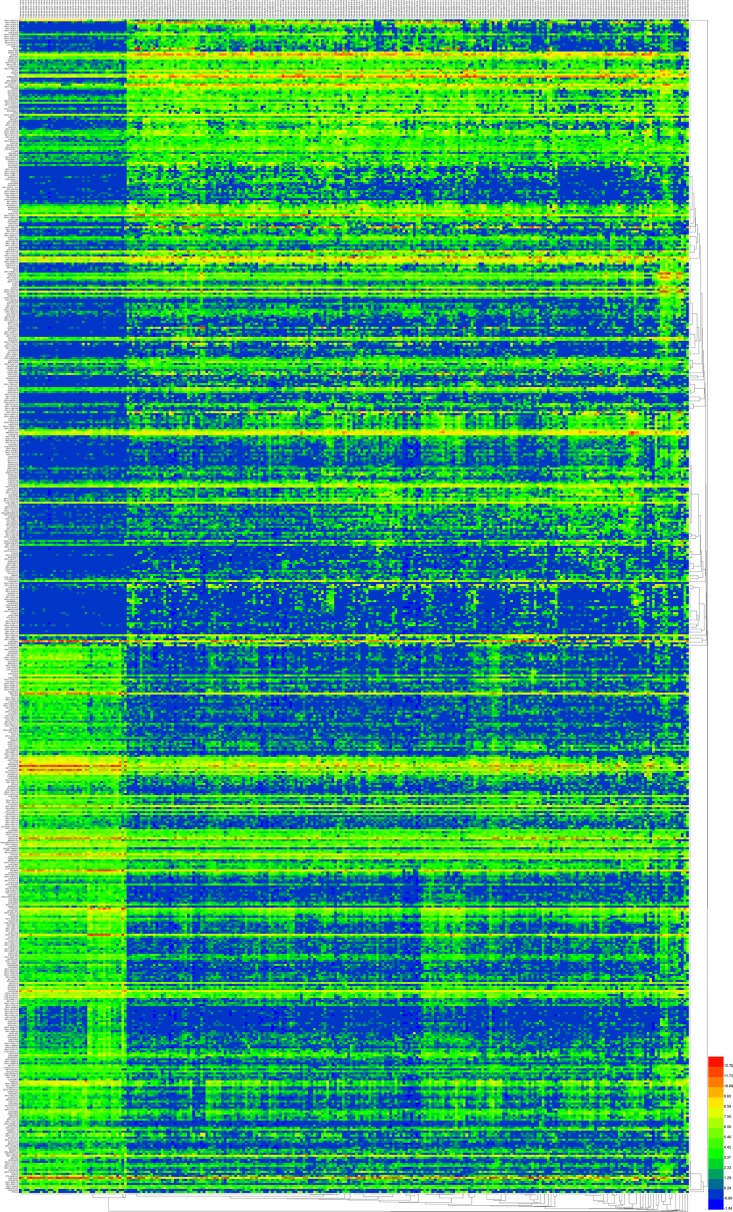
The 578 differentially expressed lncRNAs (DELs) in COAD A heatmap was drawn to show the DELs.

We initially performed differential expression analysis by comparing the expression of 7589 lncRNAs in COAD and normal colon tissue. The edgeR package identified 1430 differentially expressed lncRNAs (DELs, Figure 1A) and the DEseq package identified 584 DELs (Figure 1B). We combined these two groups of DELs together and 578 DELs showed a consistent direction of differential expression across the two methods (Figure 1C, Figure 2). Next, we excluded those cases without sufficient survival data, leaving 224 DELs that were selected for further survival analysis (Supplementary Table 1).

### Construction of the DEL-based prognostic signature

**Figure 3:**
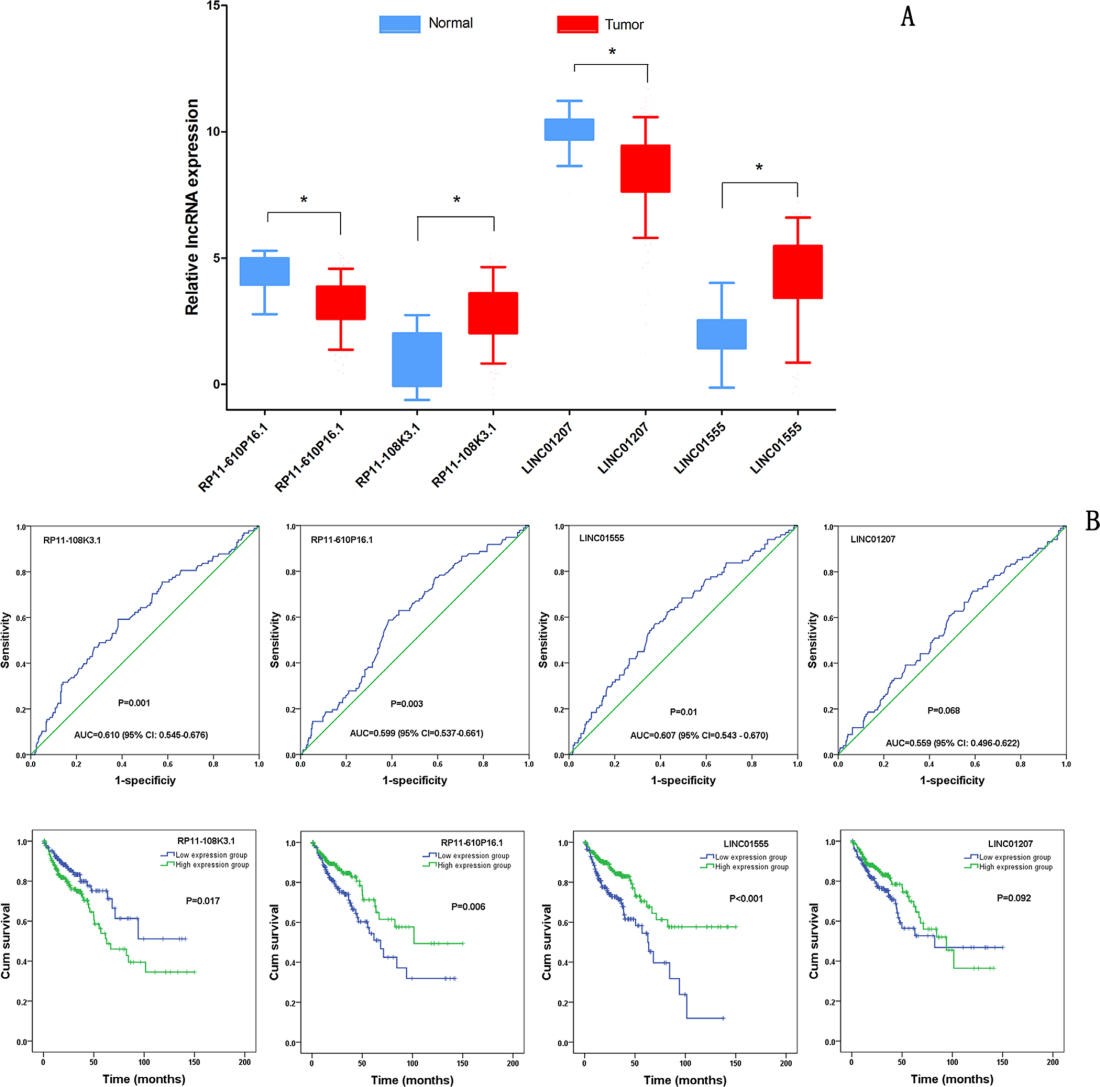
Clinical significance of four differentially expressed lncRNAs (DELs) (**A**) The expression levels of four DELs in the tumor group compared with that in the normal group; (**B**) ROC curves of the four DELs to distinguish COAD tissue from normal colon tissue; (**C**) The Kaplan-Meier curves showing the relationship between the four DELs and the overall survival. The cases were divided into a high and low expression group by the mean DEL level. *P < 0.001.

First, a univariate Cox proportional hazards regression showed that a total of 20 among the 224 DELs were identified to maintain a significant prognostic value (Table 1). Second, a multivariate Cox proportional hazards regression analysis indicated that only four DELs exhibited a significant prognostic value for COAD, including LINC01555, RP11-610P16.1, RP11-108K3.1 and LINC01207 (Figure 3). Afterwards, the risk score for predicting the OS was constructed with the formula: Risk score = exp _LINC01555_*(-0.191) + exp _RP11-610P16.1_*(-0.338) + exp _RP11-108K3.1_*(0.318) + exp _LINC01207_*(-0.163).

**Table 1:** Prognostic value of the DELs by univariate cox regression analysis

	Estimate	StdErr	ChiSq	ProbChiSq*	HazardRatio
AC016831.7	–0.209	0.065	10.521	0.001	0.811
LINC01555	–0.177	0.056	9.985	0.002	0.838
RP11-610P16.1	–0.306	0.104	8.592	0.003	0.736
AC006273.5	0.239	0.083	8.412	0.004	1.271
RP11-108K3.1	0.252	0.091	7.760	0.005	1.287
RP1-193H18.2	–0.217	0.082	7.078	0.008	0.805
LINC00675	–0.179	0.069	6.753	0.009	0.836
CTD-2619J13.17	–0.226	0.093	5.925	0.015	0.797
RP11-449D8.1	–0.154	0.064	5.838	0.016	0.857
AF064858.6	–0.154	0.064	5.791	0.016	0.857
RP11-150O12.3	–0.138	0.059	5.504	0.019	0.871
TP53TG1	–0.234	0.101	5.419	0.020	0.791
LINC00959	–0.248	0.111	4.983	0.026	0.780
SUCLG2-AS1	–0.189	0.088	4.647	0.031	0.827
LINC01315	–0.180	0.086	4.408	0.036	0.836
RP11-474D1.3	–0.066	0.032	4.340	0.037	0.936
MAFTRR	–0.184	0.088	4.331	0.037	0.832
KBTBD11-OT1	–0.172	0.084	4.174	0.041	0.842
LINC01207	–0.120	0.059	4.099	0.043	0.887
LINC01132	–0.214	0.106	4.055	0.044	0.808

* ProbChiSq equal *P*-value.

**Figure 4:**
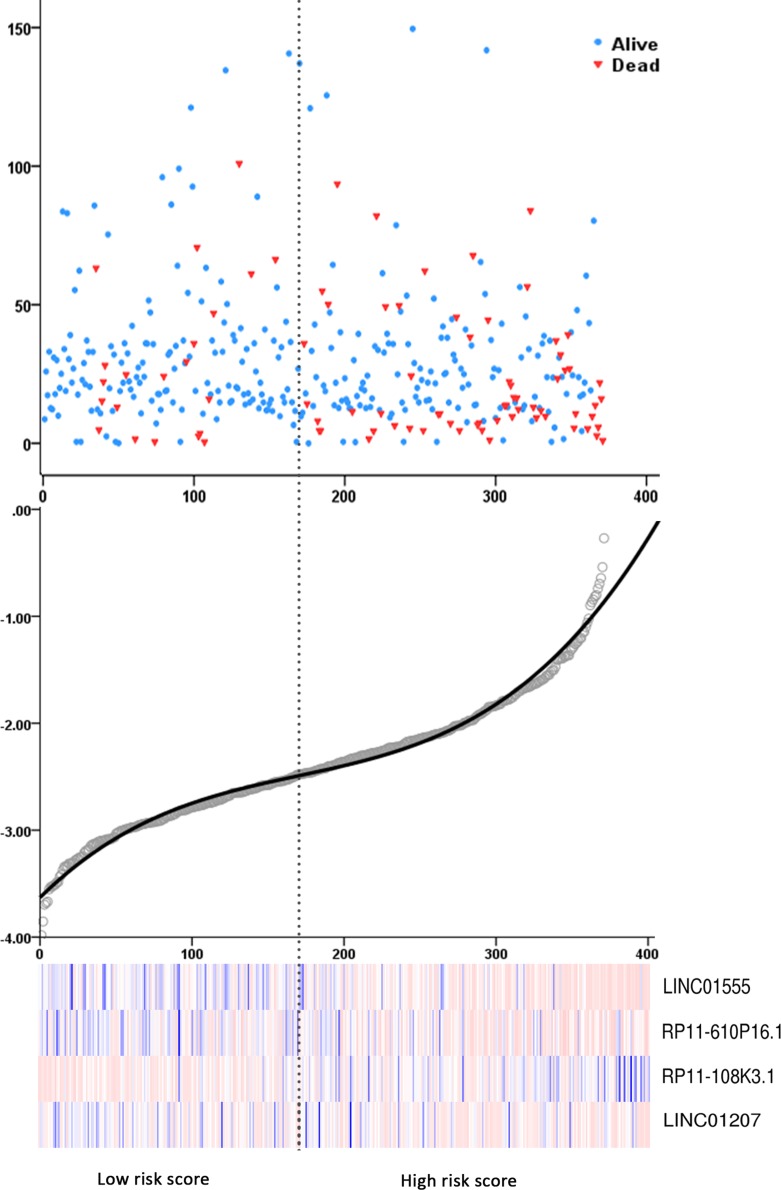
Risk score analysis of the differentially expressed lncRNA (DEL) signature of COAD (**A**) Patient survival status and time distributed by risk score; (**B**) Risk score curve of the four-DEL-signature; (**C**) Heatmap of four DELs from COAD patients. Color from blue to red indicates the expression level from low to high. The dotted line represents the individual inflection point of the risk score curve, by which the COAD patients were classified in the low-risk or high-risk group.

**Figure 5:**
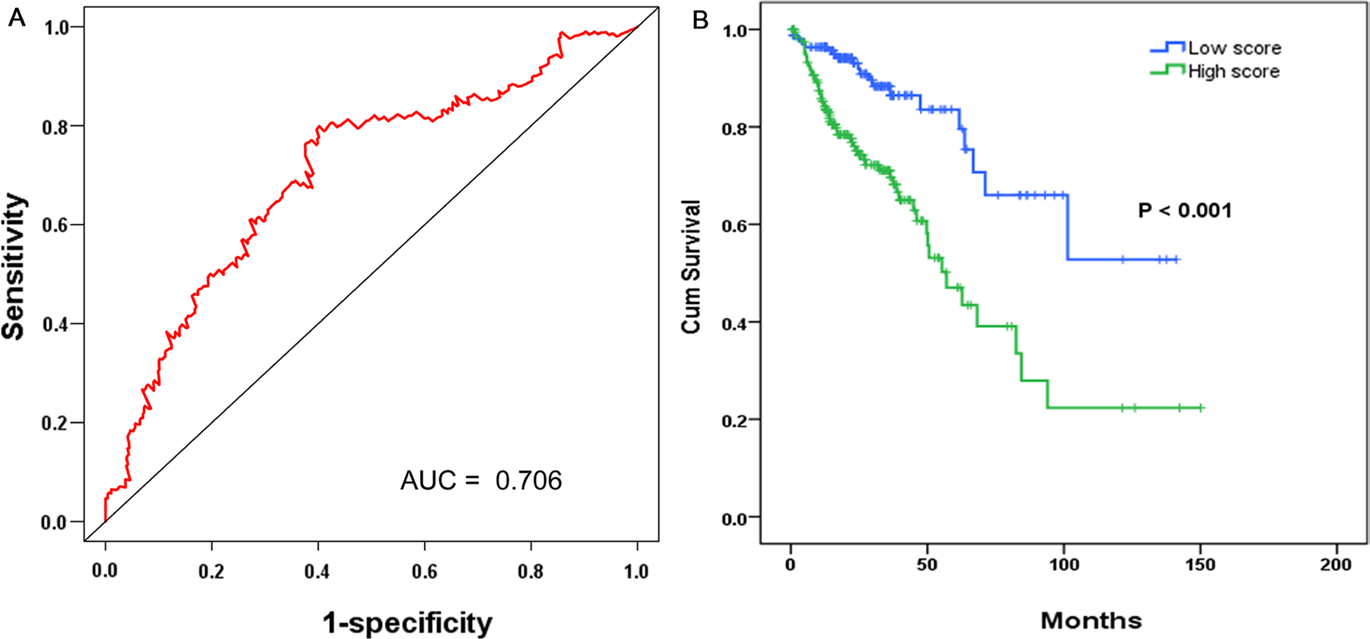
The prognostic performance of the four-differentially expressed lncRNA (DEL) signature of COAD (**A**) The prognostic performance of the risk score showed by the time-dependent receiver operating characteristic (ROC) curve for predicting the 5-years survival. (**B**) The Kaplan-Meier test of the risk score for the overall survival.

The COAD patients were divided into two groups of low-risk and high-risk based on the individual inflection point of the prognostic risk score (Figure 4). The risk score could largely predict the 5-year survival of COAD patients, as the area under ROC curve (AUC) was 0.706 (Figure 5A). Furthermore, K-M curves confirmed that the survival time of patients in the high-risk group was 72.935 ± 7.398 months, predominantly shorter than that of the low-risk group (103.402 ± 8.679 months, *P <* 0.001, Figure 5B).

**Figure 6:**
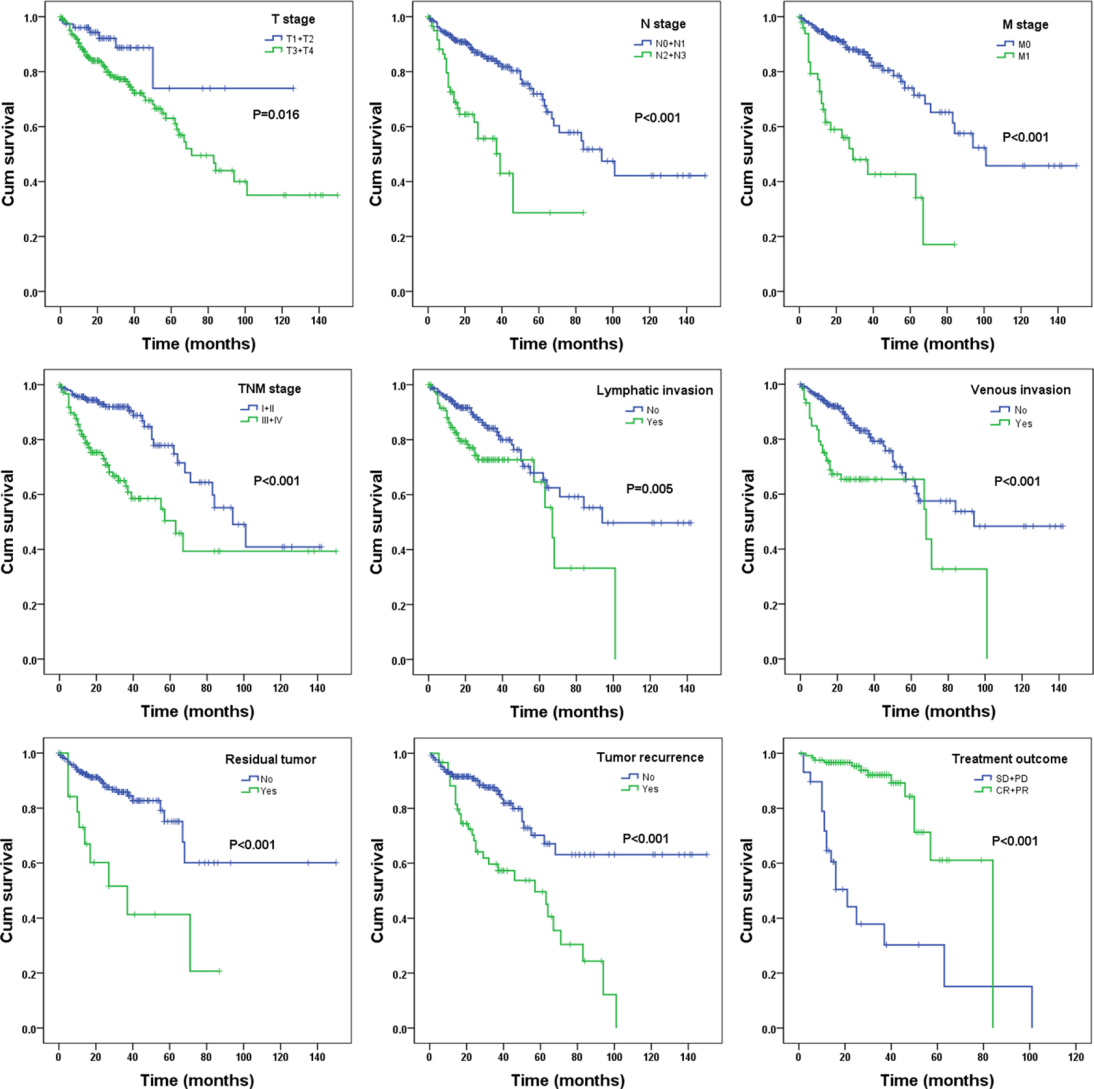
The prognostic value of different parameters for survival of COAD patients Kaplan-Meier curves of nine independent prognostic indictors, including pathologic tumor stage, pathologic node stage, pathologic metastasis, pathologic stage, lymphatic invasion, venous invasion, residual tumor, tumor recurrence and treatment outcome. CR, complete response; PR, partial response; SD, stable disease; PD, progressive disease.

Meanwhile, the prognostic value of different clinical parameters was also compared to that of the risk score. The univariate Cox proportional hazards regression showed that a number of parameters could predict poorer survival of COAD (Table 2). However, when analyzed by multivariate Cox proportional hazards regression test, only neoplasm recurrence together with the risk score from the DELs, was independent prognostic indictor of COAD (Table 2). The K-M curves of the above clinical features are shown in Figure 6.

**Table 2:** The predictive values of related clinical parameters and risk score

Variables		Patient *n* = 371	Univariate analysis		Multivariate analysis	*P*
**HR (95% CI)**	***P***	**HR (95% CI)**
Sex	Female	170	1 (reference)			
	Male	201	1.180 (0.761–1.831)	0.459		
Age	<= 65 years	162	1 (reference)			
	> 65 years	209	1.284 (0.816–2.022)	0.280		
Disease stage	I	67	1 (reference)		1 (reference)	
	II	146	2.187 (0.759–6.304)	0.147	1.225E4 (0.000–1.976E86)	0.922
	III	99	3.904 (1.354–11.257)	**0.012**	8.180E4 (0.000–1.328E87)	0.907
	IV	52	9.645 (3.351–27.758)	**< 0.001**	2.394E5 (0.000–3.875E87)	0.989
T stage	T1	10	1 (reference)			
	T2	68	0.452 (0.087–2.352)	0.345		
	T3	249	1.052 (0.256–4.332)	< 0.944		
	T4	44	4.078 (0.937–17.747)	< 0.061		
N stage	N0	227	1 (reference)		1 (reference)	
	N1	82	1.827 (1.059–3.151)	**0.030**	0.624 (0.096–4.049)	0.621
	N2-N3	61	4.348 (2.577–7.337)	**< 0.001**	0.868 (0.136–5.555)	0.881
M stage	M0	269	1 (reference)			
	M1	51	4.601 (2.773–7.636)	**< 0.001**		
Lymphatic invasion	NO	211	1 (reference)		1 (reference)	
	YES	120	1.960 (1.219–3.150)	**0.005**	0.380 (0.121–1.189)	0.096
Venous invasion	NO	248	1 (reference)		1 (reference)	
	YES	74	2.490 (1.547–4.006)	**< 0.001**	1.331 (0.438–4.048)	0.614
Treatment outcome	CR+PR	123	1 (reference)			
	SD+PD	29	7.320 (3.491–15.347)	**<0.001**		
Radiotherapy	NO	308	1 (reference)			
	YES	8	0.740 (0.102–5.355)	0.765		
Neoplasm recurrence	NO	256	1 (reference)		1 (reference)	
	YES	60	2.990 (1.855–4.819)	**< 0.001**	3.030 (1.234–7.440)	**0.016**
Residual tumor	R0	263	1 (reference)		1 (reference)	
	R1+R2	20	3.953 (1.933–8.082)	**< 0.001**	1.660 (0.607–4.537)	0.323
Dimession	<=10 mm	137	1 (reference)			
	> 10 mm	124	0.944 (0.537–1.659)	0.840		
Risk score	Low	170	1 (reference)		1 (reference)	
	High	201	2.948 (1.779 – 4.886)	**< 0.001**	9.389 (2.737–32.213)	**<0.001**

HR: hazard ratio; CI: confidence interval; SD: stable disease; PD: progressive disease; CR: complete response; PR: partial response.

**Figure 7:**
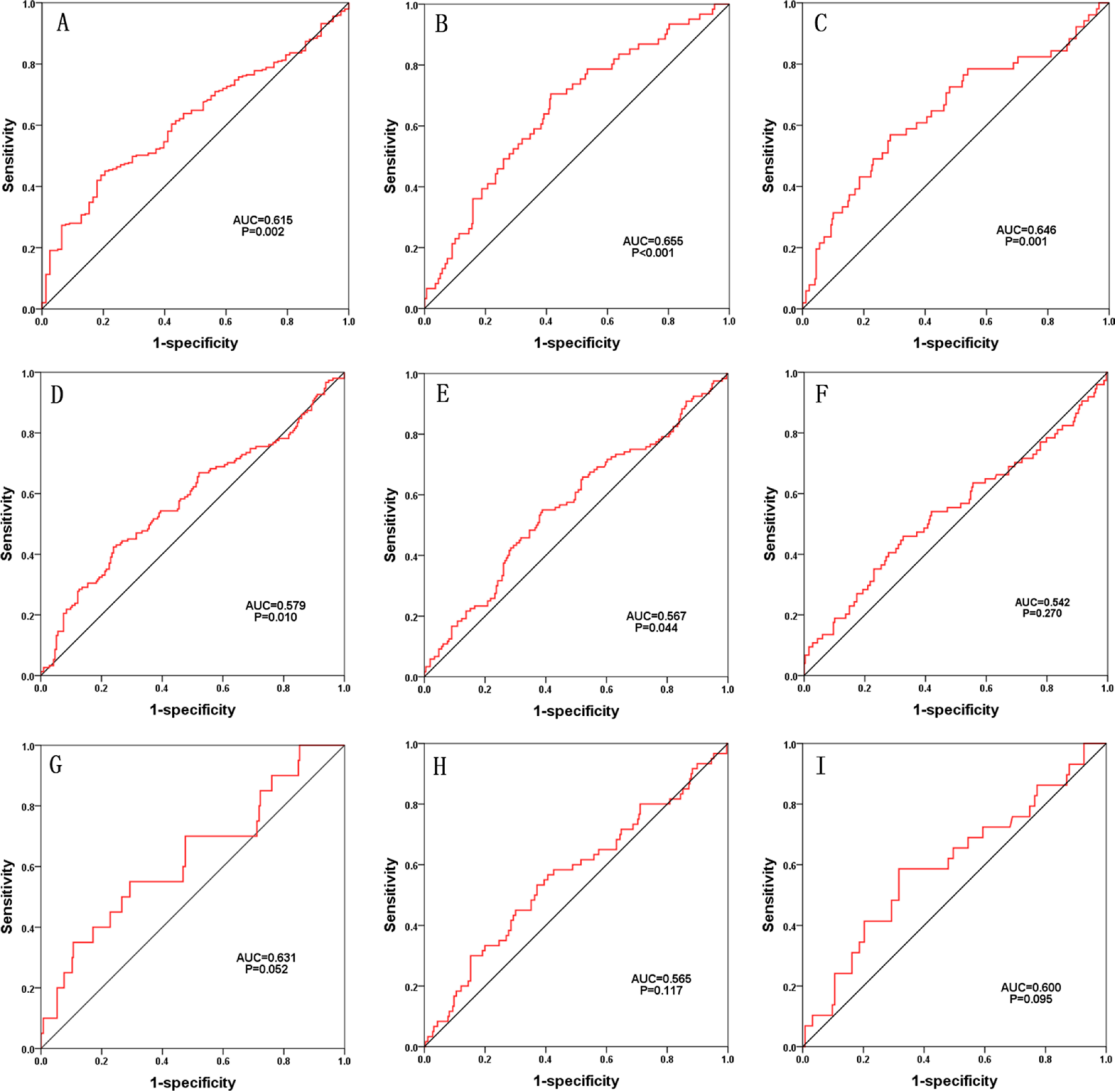
The predictive value of the risk score for the clinical status characteristic (ROC) curve predicting the different clinical parameters: pathologic tumor stage, pathologic node stage, pathologic metastasis, pathologic stage, lymphatic invasion, venous invasion, residual tumor, tumor recurrence and treatment outcome.

**Figure 8:**
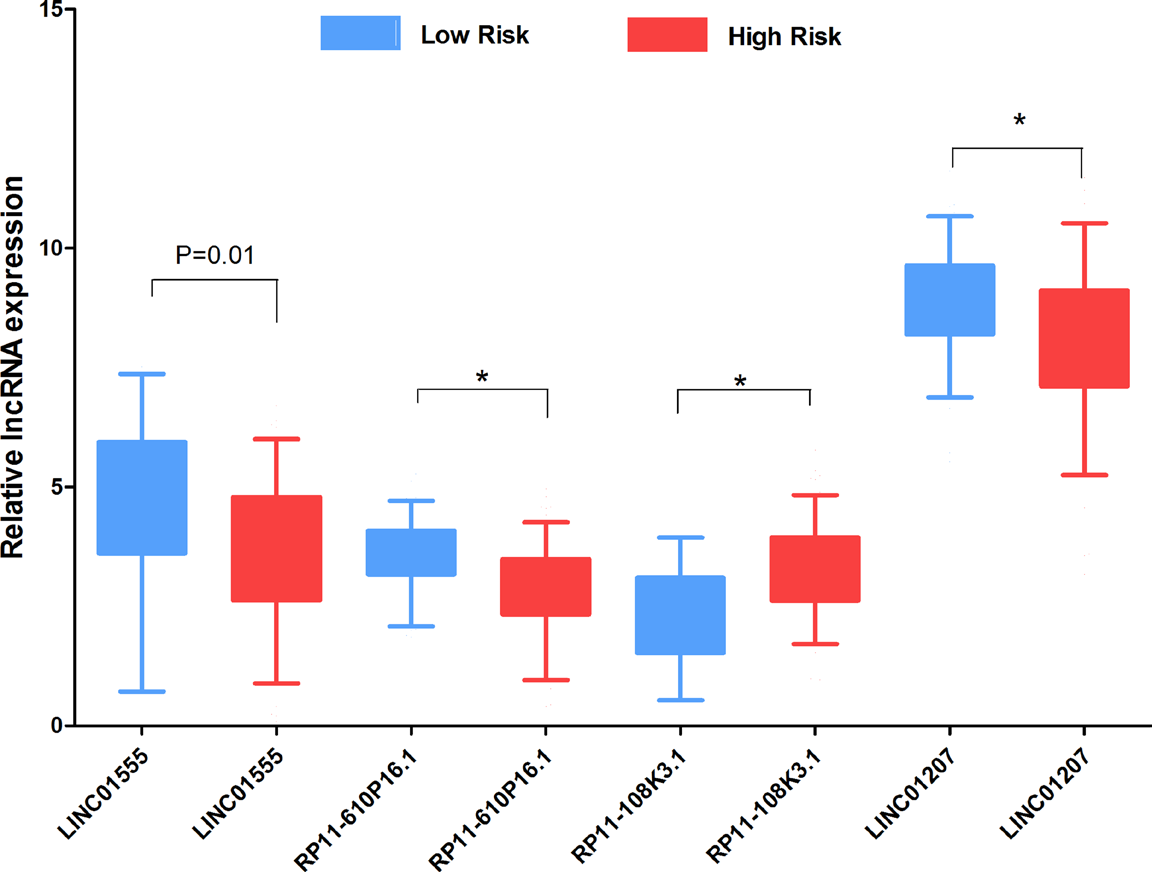
The expression level of the four lncRNAs in the low- and high-risk groups The difference in the expression level of LINC01555, RP11-610P16.1, RP11-108K3.1 and LINC01207 between the low-risk group and high-risk group. *P < 0.001.

We also assessed the relationship between the risk score based on the DELs signature and various clinical features, and the risk score showed moderate prognostic value for predicting the status of tumor stage, metastasis and lymphatic invasion (Figure 7). The expression pattern of these four DELs in the low- and high-risk group is also presented in Figure 8.

### Validation of the four-DEL-signature in READ

**Figure 9:**
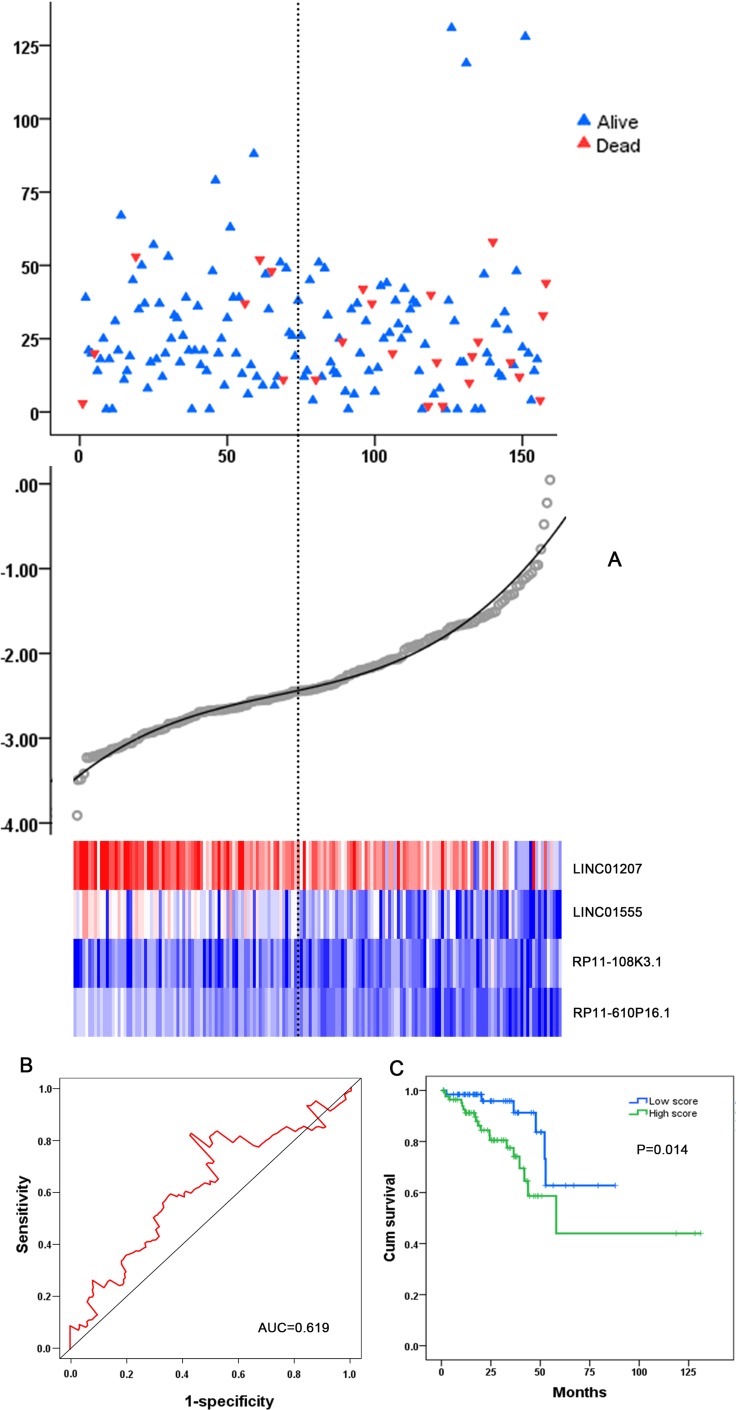
Validation of the four-DEL-signature in READ (**A**) READ patients were divided into high- and low-risk groups using the risk score generated from the four-DEL-signature. The relationship between survival and risk score is presented (top); The risk score curve is drawn to classify the READ patients into low- and high- risk groups (middle); The risk scores of the READ patients are shown in a heatmap (bottom). (**B**) ROC curve for predicting 5-year survival in READ patients by the risk score. (**C**) Kaplan-Meier curves of the four-lncRNA signature for READ patients.

For validation, the READ patients were also divided into low-risk and high-risk groups on the basis of the prognostic risk score (Figure 9A). Though the AUC of ROC to predict 5-year survival was slightly less than that in COAD patients (Figure 9B), K-M curves did show a close relationship between the four-DEL-signature and survival (*P* = 0.014, Figure 9C). Additionally, both univariate (HR: 3.006, 95% CI: 1.192 - 7.586, *P* = 0.020) and multivariate Cox proportional hazards regression tests (HR: 8.602, 95% CI: 1.159–63.839, *P* = 0.035) revealed that the risk score of four-DEL-signature was an independent prognostic indictor of READ (Supplementary Table 2).

### Functional assessment of the prognostic DELs

**Figure 10:**
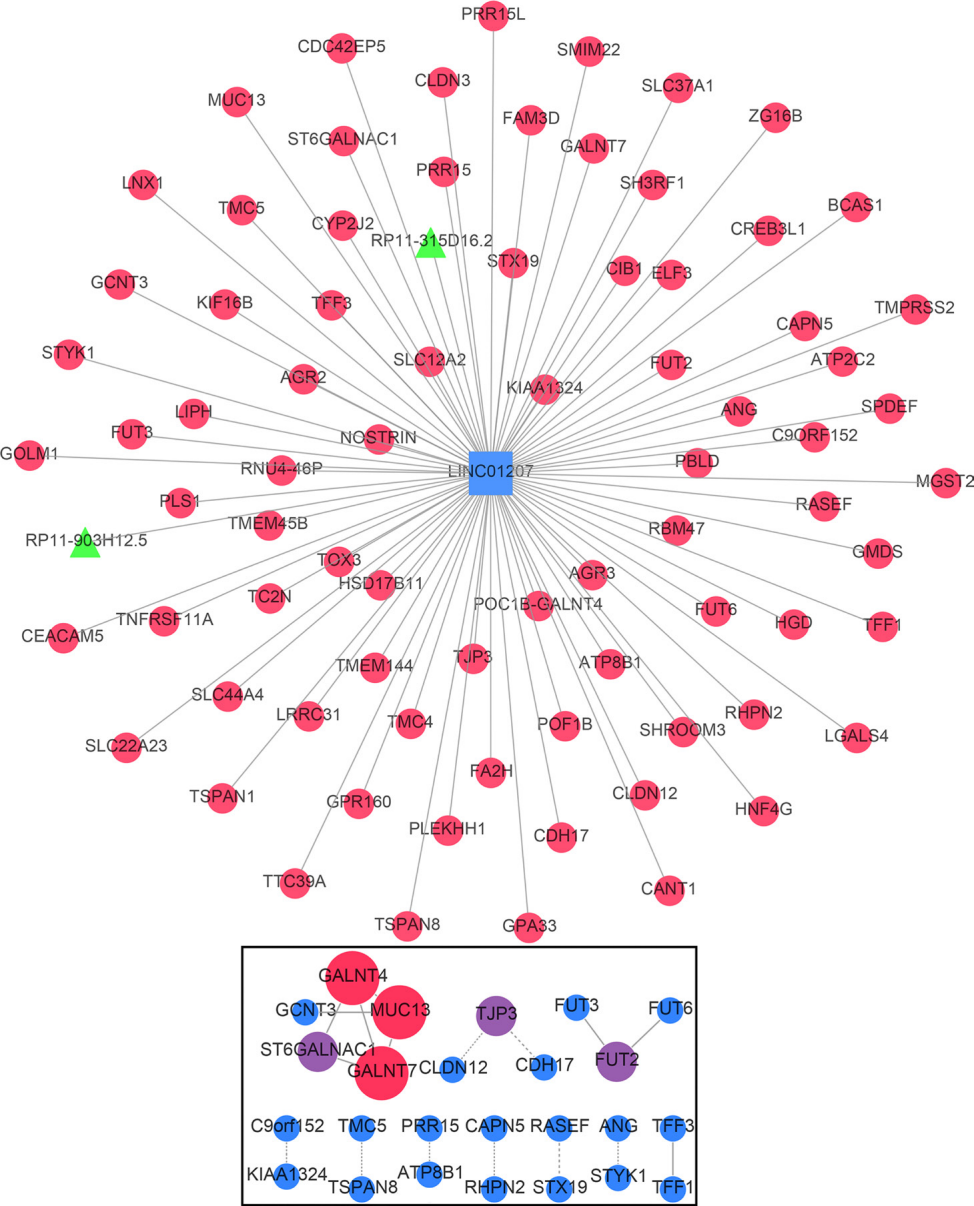
Gene network of the correlative genes of LINC01207 The network of genes co-expressed with LINC01207. LncRNAs are shown as green nodes and mRNAs as red nodes. We measured LINC01207 co-expression using the Multi Experiment Matrix (MEM) software. The Affymetrix GeneChip Human Genome U133 Plus 2.0 platform type was selected for further analysis. The top 100 genes were used to draw the network schematic. The bottom box displays the protein-protein interaction (PPI) network of related genes.

**Figure 11:**
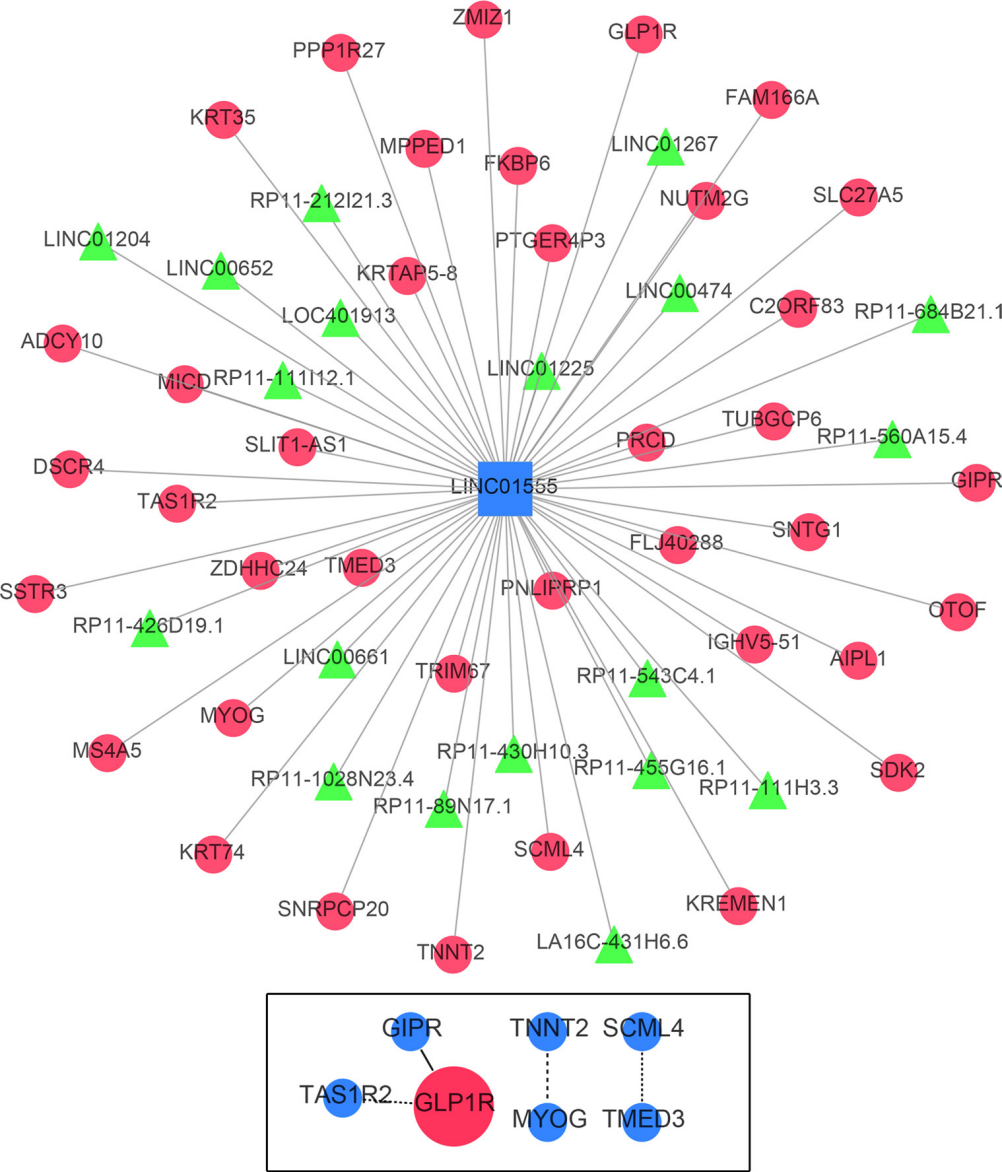
Gene network of the correlative genes of LINC01555 The network of genes co-expressed with LINC01555. LncRNAs are shown as green nodes and mRNAs are shown as red nodes. We detected the co-expression of LINC01555 using Multi Experiment Matrix (MEM) software. The Affymetrix GeneChip Human Genome U133 Plus 2.0 platform type was applied for further examination. The top 100 genes were used to draw the network schematic. The bottom box displays the protein-protein interaction (PPI) network of related genes.

**Figure 12:**
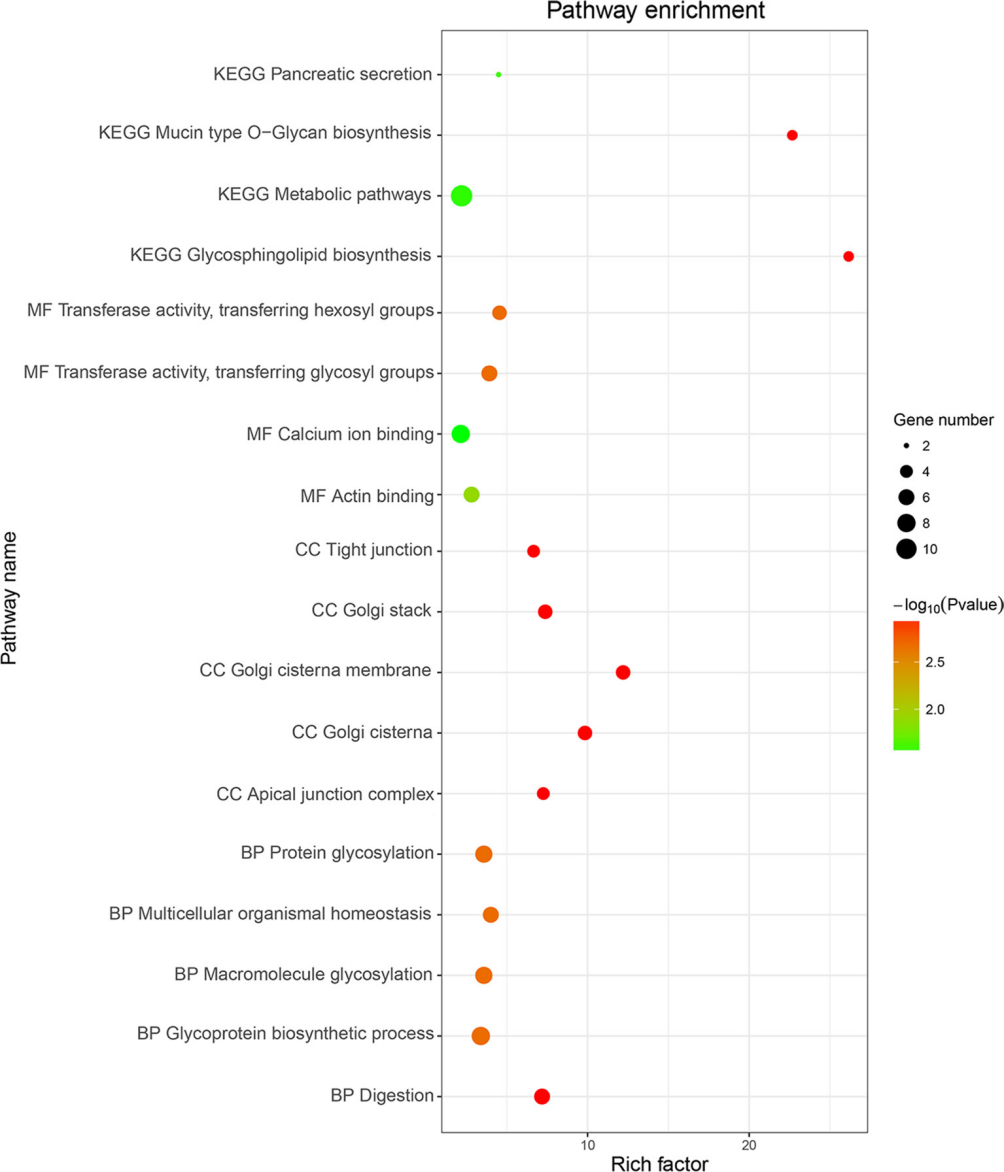
GO and KEGG term analysis of potential genes related to LINC01555 and LINC01207 The Rich factor shows the degree of enrichment, which was calculated by the formula: (the number of selected genes in a term/total number of selected genes)/(the total number of genes in a term of the database/ the total number of genes in the database). The Node size represents the number of selected genes, and color represents the *p-value* of the enrichment analysis. CC, cellular component; MF, molecular function; BP, biological process.

Only correlative genes for LINC01555 and LINC01207 could be determined using the Multi-Experiment Matrix (MEM, Figure 10 and Figure 11). Forty genes were collected for LINC01555 and 78 genes for LINC01207. Using kyoto encyclopedia of genes and genomes (KEGG) pathway analysis, the correlative genes of LINC01555 were found to be enriched in the cAMP signaling pathway and the neuroactive ligand-receptor interaction pathways, while genes related to LINC01207 were enriched in mucin type O-Glycan biosynthesis pathway, the - lacto/neolacto-series glycosphingolipid biosynthesis pathway and metabolic pathway. Similarly, some Gene Ontology (GO) terms were also enriched (Table 3, Figure 12). For construction of the protein-protein interaction (PPI) network, there were 3 genes with more than 3 nodes for LINC01207 (GALNT4, GALNT7, MUC13), which were regarded as hub genes. However, we failed to observe similar hub genes for LINC01555.

**Table 3:** GO terms and pathway analyses with the correlative genes of LINC01555 and LINC01207

Category	Term	*P* value	Genes
**LINC01555**			
KEGG pathway	cAMP signaling pathway	0.016	GIPR, ADCY10, GLP1R
	Neuroactive ligand-receptor interaction	0.029	SSTR3, GIPR, GLP1R
Biological process	Meiotic nuclear division	0.034	TUBGCP6, FKBP6
	Regulation of heart contraction	0.043	TNNT2, GLP1R
	Cellular response to estradiol stimulus	0.049	SSTR3, MYOG
Cellular component	Basal part of cell	0.021	OTOF, ADCY10
Molecular function	Structural molecule activity	0.047	KRT74, SNTG1, KRT35
**LINC01207**			
KEGG pathway	Mucin type O-Glycan biosynthesis	< 0.001	GCNT3, GALNT7, POC1B-GALNT4, ST6GALNAC1
	Glycosphingolipid biosynthesis - lacto and neolacto series	0.003	FUT6, FUT3, FUT2
	Metabolic pathways	0.007	GCNT3, CYP2J2, GALNT7, GMDS, POC1B-GALNT4, FUT6, FUT3, HGD, FUT2, ST6GALNAC1
Biological process	Protein glycosylation	< 0.001	GALNT7, POC1B-GALNT4, FUT6, FUT3, FUT2, ST6GALNAC1
	L-fucose catabolic process	0.001	FUT6, FUT3, FUT2
	Fucosylation	0.001	FUT6, FUT3, FUT2
	Maintenance of gastrointestinal epithelium	0.001	TFF1, MUC13, PBLD
	O-glycan processing	0.002	GCNT3, GALNT7, POC1B-GALNT4, MUC13
	Carbohydrate metabolic process	0.005	GCNT3, GALNT7, POC1B-GALNT4, TFF1, FUT2
	Positive regulation of establishment of protein localization to plasma membrane	0.006	PLS1, AGR2, CIB1
	Lung goblet cell differentiation	0.012	SPDEF, AGR2
	Oligosaccharide biosynthetic process	0.042	FUT3, ST6GALNAC1
Cellular component	Extracellular exosome	< 0.001	GCNT3, TSPAN1, CYP2J2, GALNT7, CLDN3, SLC44A4, POC1B-GALNT4, FUT6, KIAA1324, TSPAN8, PBLD, CANT1, ZG16B, ANG, PLS1, FUT3, TFF3, CEACAM5, FUT2, MUC13, GOLM1, CIB1, BCAS1, TMPRSS2, CAPN5, SLC12A2, GMDS, TMC5, TMC4, HGD, GPA33
	Golgi cisterna membrane	0.003	FUT6, FUT3, FUT2, CANT1
	Golgi apparatus	0.006	SH3RF1, POC1B-GALNT4, FUT6, KIAA1324, ATP8B1, FUT3, FUT2, GOLM1, ST6GALNAC1, CIB1
	Integral component of plasma membrane	0.008	TMPRSS2, ATP2C2, TNFRSF11A, TSPAN1, SLC12A2, CLDN3, SLC22A23, KIAA1324, ATP8B1, GPA33, TSPAN8, CEACAM5, GOLM1
	Integral component of membrane	0.009	GCNT3, GPR160, FAM3D, GALNT7, CLDN3, SLC44A4, POC1B-GALNT4, FUT6, KIAA1324, CLDN12, CANT1, TMEM144, STX19, ATP8B1, FUT3, CREB3L1, FUT2, MUC13, TMPRSS2, TMEM45B, SLC12A2, FA2H, TMC5, SLC22A23, TMC4, ST6GALNAC1, STYK1, ATP2C2, CDH17, SMIM22, MGST2
	Bicellular tight junction	0.010	CLDN3, POF1B, CLDN12, TJP3
	Golgi membrane	0.028	ATP2C2, GCNT3, GALNT7, POC1B-GALNT4, FUT6, FUT3, ST6GALNAC1
	Apical plasma membrane	0.030	SHROOM3, SLC12A2, ATP8B1, MUC13, CIB1
	Perinuclear region of cytoplasm	0.040	SH3RF1, ATP2C2, TSPAN1, RASEF, POC1B-GALNT4, RHPN2, CIB1
	Organelle membrane	0.044	CYP2J2, FA2H, MGST2
Molecular function	Fucosyltransferase activity	< 0.001	FUT6, FUT3, FUT2
	3-galactosyl-N-acetylglucosaminide 4-alpha-L-fucosyltransferase activity	0.011	FUT6, FUT3
	Alpha-(1->3)-fucosyltransferase activity	0.030	FUT6, FUT3
	Dystroglycan binding	0.034	AGR3, AGR2
	carbohydrate binding	0.040	ZG16B, GALNT7, LGALS4, POC1B-GALNT4

## DISCUSSION

In the current study, to define lncRNAs significantly related to OS, a univariate Cox proportional hazards regression with the significance level set at 0.05 was first performed on 7589 lncRNAs from 371 COAD patients according to the defined criterion in a large number of COAD patients based on the data downloaded from the TCGA database. A total of four lncRNAs were identified. We then developed a risk score by combining the four lncRNAs and found that this four-lncRNA signature could independently predict OS in COAD patients, which was further validated in READ patients. As far as we know, this is the first study to construct a risk score by mining TCGA data for the survival assessment of colorectal cancer patients.

Colorectal cancer is one of the deadliest solid malignancies, and the involvement of noncoding RNAs in the development, diagnosis, and prognosis of colorectal cancer has been widely investigated [33–35]. In addition to the aggressive properties of colorectal cancer, the lack of specific biomarkers for its diagnosis, therapeutic effect monitoring and prognosis might also be responsible for the low survival rate. Hence, there is a critical need for reliable prognostic factors pinpointing a poor outcome. Recent large-scale genomic analyses have made it possible to reveal a catalogue of molecular characteristics associated with colorectal cancer outcome [44, 46–53]. However, most of the existing studies have focused on mRNA and microRNA expression [54–56]. Knowledge is now rapidly emerging on the functional roles of lncRNAs in cancer initiation and progression, representing a significant untapped molecular resource for cancer prognosis as well.

The relationship between aberrant lncRNAs and survival of colorectal cancer has been studied in small samples using distinct approaches. Li et al. [57] analyzed the prognostic value of 21 lncRNAs by PCR array in 30 colorectal cancer patients and reported that higher levels of AFAP1-AS1, BCAR4, H19, HOXA-AS2, MALAT1 or PVT1 and a lower level of ADAMTS9-AS2 could predict a poor prognosis of colorectal cancer patients. Similarly, Wang et al. [58] studied lncRNA expression profiling using microarray in six cases of colorectal cancer patients. Multivariate Cox analysis revealed that lncRNA NR_029373 and NR_034119 were both independently related to the disease-specific survival rate. Furthermore, Sun et al. [25] searched in GEO datasets and achieved five studies: GSE8671, GSE22598, GSE23878, GSE9348, and GSE37364, that studied lncRNAs in 150 cases of colorectal cancer patients. They found that one lncRNA, AK098081 could be considered an independent risk factor for colorectal cancer patients (HR = 1.896, 95% CI = 1.393–2.579, *P <* 0.001). Surprisingly, no consistent lncRNA has been verified by different groups, potentially due, at least in part, to the limited sample size and differing detection methods. Compared with previous studies, our study uses data from the TCGA database with high-throughput analysis of lncRNAs from a larger sample size. Herein, we report that expression of four novel lncRNAs (LINC01555, RP11-610P16.1, RP11-108K3.1 and LINC01207) could also become a new independent risk factor for colorectal cancer patients. Moreover, the risk score constructed from these four lncRNAs could be an indicator for the colorectal cancer patients in the clinical setting.

No study as of yet has investigated the function of the aforementioned four lncRNAs. Here, we performed MEM to gather the correlative genes of these four lncRNAs. However, the correlative genes were only found for LINC01555 and LINC01207 in this step. Interestingly, the correlative genes of LINC01555 were enriched in cAMP signaling pathway and neuroactive ligand-receptor interaction pathway, whereas the genes correlative to LINC01207 were enriched in mucin type O-Glycan biosynthesis pathway, – the lacto/neolacto-series glycosphingolipid biosynthesis pathway and metabolic pathway, which are all classical signaling pathways closely related to the tumorigenesis and progression of malignancies. For instance, intracellular cAMP has been proposed to impact the biological behavior, namely to suppress the growth of colorectal cancer cells [59, 60]. Most likely, LINC01555 could play substantial roles in the tumorigenesis and development of colorectal cancer via influencing cAMP signaling pathway. Therefore, the functional enrichment analysis may offer a clue for elucidating the role of LINC01555 and LINC01207 in carcinogenesis of colorectal cancer and the specific underlying molecular mechanisms. However, since the research on the clinical and biological function of LINC01555, RP11-610P16.1, RP11-108K3.1, and LINC01207 is still nonexistent in colorectal cancer patients, there is a lot of research that needs to be accomplished.

The findings of the current study may have substantial clinical significance or implications; however, some limitations should be considered. First, the mean time of follow-up was 29.375 months for COAD and 26.965 months for READ patients, and a study including a longer follow-up time is warranted to validate our findings in the future. Second, the data from TCGA were based on the RNA-seq technique; other experimental methods are needed to verify the current finding. Third, the roles of LINC01555, RP11-610P16.1, RP11-108K3.1, and LINC01207 in colorectal cancer are unknown; *in vitro* and *in vivo* experiments are expected to answer this question.

In conclusion, by analyzing the genome-wide lncRNA expression profiles in a large cohort from TCGA, we identified a four-lncRNA signature, which could act as an indicator for patient outcome and could be a potential independent biomarker for prognosis prediction of colorectal cancer. We will gather clinical samples and validated these findings experimentally in our future work.

## MATERIALS AND METHODS

### Differentially expressed lncRNAs

RNA sequencing (RNA-Seq) data from 521 individuals with COAD were obtained from TCGA data portal (https://tcga-data.nci.nih.gov/docs/publications/tcga/?), including data from 480 COAD tissue samples and 41 non-tumorous adjacent-normal colon tissue samples up to November 9, 2016. Since the data were provided by TCGA, additional approval by an ethics committee was not needed. Data processing was performed in line with the TCGA human subject protection and data access policies. This dataset consisted of called gene counts for 60,244 mRNAs, which were assessed on the IlluminaHiSeq RNA-Seq platform. In the current paper, only lncRNAs with description from NCBI or Ensemble were selected for further study. Finally, we obtained the expression profiles of 7581 lncRNAs. We then filtered the differentially expressed lncRNAs (DELs) using two individual R packages: edgeR [61, 62] and DEseq [63, 64], with Padj < 0.05 and logFC > 1 of expression level between comparison of tumor and adjacent normal colon tissue. Since a strategy that combines edgeR and DESeq is proposed for large sample sizes from TCGA [65], here in the current study, this combination of edgeR and DESeq was adopted. The overlapping DELs obtained using both edgeR and DEseq were sent for further survival analysis. For validation, the relevant data including lncRNA levels and clinicopathological parameters were also downloaded for 158 READ tissues and 10 non-tumorous controls.

### Construction of the DEL-based prognostic signature and statistical analysis

The DELs that were 0 in greater than 10% of all subjects were eliminated. The expression level of each DEL was log2-transformed for further analysis. Meanwhile, clinicopathological parameters and survival data were also downloaded from TCGA. The subjects without clinical data were excluded, which resulted in a 371-sample cohort with 224 DELs enrolled in the survival analysis (Supplementary Table 1). The end-point in our study was OS. The average follow-up time was 29.375 months in this COAD cohort and 26.965 months in READ cases.

The univariate Cox proportional hazards regression with a significance level set at 0.05 was performed to obtain the DELs that are closely correlated with the OS. A total of four DELs were identified. The multivariate cox regression model was further performed to test the prognostic value of the DELs. A prognosis risk score for predicting OS was established on the basis of a linear combination of the expression level multiplied by the regression coefficient derived from the multivariate cox regression model (β) with the following formula as previously reported:

Risk score = exp_DEL1_*β_DEL1_ + exp_DEL2_*β_DEL2_ + … exp_DELn_*β_DELn_.

COAD and READ patients were divided into two groups: high-score and low-score, with the cut-off of the individual inflection point of the prognostic risk score [66]. Univariate Cox proportional hazards regression analyses were further conducted to investigate the effects of various clinical characteristics and the risk score on the OS of COAD and READ patients. Each predictor identified via the univariate analysis was further evaluated by a multivariate Cox proportional hazards regression analysis to determine whether the lncRNA prognostic model was independent of other clinical variables, adjusting for age, tumor stage, grade, surgical debulking status, and risk scores. Hazard ratio (HR) and 95% confidence intervals (CIs) were assessed. The time-dependent receiver operating characteristic (ROC) curve analysis within 5 years as the defining point was also performed using the R package “survivalROC”, to evaluate the predictive accuracy of the prognostic model for time-dependent disease outcomes [67]. Kaplan-Meier survival curves were used to estimate the OS time for COAD and READ patients with predicted high- or low-risk scores, and the survival differences between the high-risk group and low-risk group were assessed by a two-sided log-rank test using SPSS 22.0 software (SPSS Inc., Chicago, IL, USA) [68].

The relationship between the DEL signature and clinical features were examined using a Chi-square test. An ROC curve was drawn to assess the predictive significance of the risk score for the patient outcome after the first course of treatment. If a two-sided *P-value* was less than 0.05, statistical significance was determined. The statistical analyses were conducted with SPSS22.0 software (SPSS Inc., Chicago, IL, USA).

### Functional assessment of prognostic DELs

The correlative genes of the DELs were collected using the Multi-Experiment Matrix (MEM) (http://biit.cs.ut.ee/mem/index.cgi) [69]. Given a gene as an input, the MEM ranks other genes by similarity in each separable data set. In essence, this analysis is a new rank aggregation method that takes the individual rankings and determines a score of significance, and then, a ranking across all datasets at the same time. Functional enrichment analysis at the GO and KEGG pathway levels and PPI assessments were employed to infer the lncRNA function with the DAVID Bioinformatics Tool (https://david.ncifcrf.gov/, version 6.7) and STRING database. The enriched GO terms and the KEGG pathways with *p-value* < 0.05 were regarded as potential function of the prognostic lncRNAs. The DELs and the correlative genes were visualized as a network using Cytoscape. A selection of protein pairs from the PPI assessment with an association score greater than 0.4 and a number of nodes beyond 3 were the final products of the correlative genes.

## SUPPLEMENTARY FIGURE AND TABLES





## Abbreviations

lncRNAs: long non-coding RNAs
COAD: colon adenocarcinoma
READ: rectal adenocarcinoma
OS: overall survival
TCGA: The Cancer Genome Atlas
DELs: differentially expressions lncRNAs
MEM: Multi-Experiment Matrix
RNA-Seq: RNA sequencing
HR: hazard ratio
ROC: receiver operating characteristic
GO: Gene ontology
KEGG: Kyoto encyclopedia of genes and genomes
PPI: protein-protein interaction
SDV: standard deviation
CR: complete response
PR: partial response
SD: stable disease
PD: progressive disease
AUC: area under the curve
CI: confidence interval
