# Supplementary material for: Comprehensive investigation of a novel differentially expressed lncRNA expression profile signature to assess the survival of patients with colorectal adenocarcinoma
Source: Oncotarget. 2017 Feb 6;8(10):16811–28. doi: 10.18632/oncotarget.15161 (PMC5370003; doi:10.18632/oncotarget.15161)
Supplement: Supplementary file 1 [file oncotarget-08-16811-s001.pdf]

Comprehensive investigation of a novel differentially expressed lncRNA expression profile signature to assess the survival of patients with colorectal adenocarcinoma

Supplementary Materials

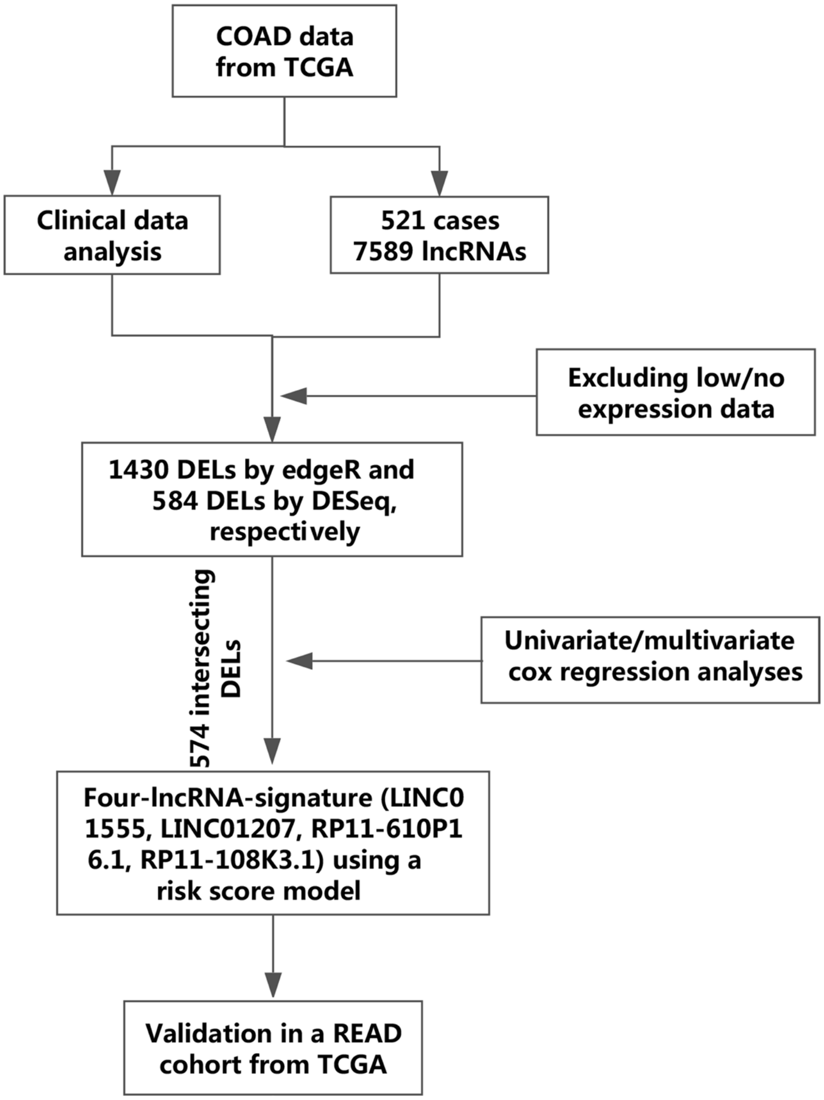

Supplementary Figure 1: Flow chart of our study. TCGA: The Cancer Genome Atlas; COAD: Colon Adenocarcinoma; READ: Rectal Adenocarcinoma; DELs: Differentially expressed lncRNAs.

Supplementary Table 1: Prognosis analysis of 224 differentially expressed lncRNAs in COAD.
See Supplementary_Table_1

Supplementary Table 2: The predictive values of related clinical parameters and risk score in READ

Variables		Patient	Univariate analysis		Multivariate analysis	
		***n* = 158**	**HR (95% CI)**	***P***	**HR (95% CI)**	***P***
Sex	Female	70	1 (reference)			
	Male	88	0.862 (0.392–1.897)	0.713		
Age	<=65 years	79	1 (reference)		1 (reference)	
	>65 years	79	4.425 (1.654–11.841)	**0.003**	33.434 (3.233–345.722)	**0.003**
Disease stage	I	30	1 (reference)			
	II	48	1.003 (0.182–5.527)	0.997		
	III	48	2.356 (0.490–11.326)	0.285		
	IV	24	5.885 (1.238–27.969)	**0.026**		
T stage	T1	9	1 (reference)			
	T2	28	13958.374 (0.000–4.565E+83)	0.919		
	T3	107	11386.296 (0.000–3.717E+83)	0.920		
	T4	13	43000.631 (0.000–1.406E+84)	0.909		
N stage	N0	81	1 (reference)		1 (reference)	
	N1	42	2.419 (0.841- 6.958)	0.101	0.570 (0.054–6.023)	0.641
	N2-N3	32	3.685 (1.390–9.766)	**0.009**	11.981 (2.018–71.122)	**0.006**
M stage	M0	119	1 (reference)			
	M1	23	3.390 (1.415–8.121)	**0.006**		
Lymphatic invasion	NO	83	1 (reference)			
	YES	58	1.292 (0.542–3.076)	0.563		
Venous invasion	NO	103	1 (reference)			
	YES	35	1.869 (0.773–4.520)	0.165		
Treatment outcome	CR+PR	40	1 (reference)			
	SD+PD	7	4.781 (0.296–77.286)	0.270		
Radiotherapy	NO	110	1 (reference)			
	YES	22	0.035 (0.000–5.018)	0.186		
Neoplasm recurrence	NO	60	1 (reference)			
	YES	81	1.680 (0.620–4.549)	0.307		
Residual tumor	R0	117	1 (reference)		1 (reference)	
	R1+R2	13	4.839 (1.834–12.763)	**0.001**	7.337 (1.207–44.582)	**0.030**
Dimession	<=10mm	73	1 (reference)			
	> 10mm	56	4.204 (1.176–15.032)	**0.027**		
Risk score	Low	68	1 (reference)		1 (reference)	
	High	90	3.006 (1.192–7.586)	**0.020**	8.602 (1.159–63.839)	**0.035**

HR: hazard ratio; CI: confidence interval; SD: stable disease; PD: progressive disease; CR: complete response; PR: partial response.
