# Supplementary material for: Comprehensive investigation of a novel differentially expressed lncRNA expression profile signature to assess the survival of patients with colorectal adenocarcinoma
Source: Oncotarget. 2017 Feb 6;8(10):16811–28. doi: 10.18632/oncotarget.15161 (PMC5370003; doi:10.18632/oncotarget.15161)
Supplement: Supplementary file 2 [file oncotarget-08-16811-s002.doc]

**Supplementary table 1 Prognosis analysis of 224 differentially expressed lncRNAs in COAD**

| ID | Estimate | StdErr | ChiSq | ProbChiSq | HazardRatio |
| --- | --- | --- | --- | --- | --- |
| AC016831.7 | -0.209489179 | 0.064585555 | 10.52088802 | 0.001180328 | 0.810998415 |
| LINC01555 | -0.176932292 | 0.055992195 | 9.985256581 | 0.001577986 | 0.837836511 |
| RP11-610P16.1 | -0.306205749 | 0.104462494 | 8.592230109 | 0.003376003 | 0.736235125 |
| AC006273.5 | 0.23944622 | 0.082560052 | 8.411550527 | 0.003728445 | 1.270545353 |
| RP11-108K3.1 | 0.25248926 | 0.090636907 | 7.760249152 | 0.005340859 | 1.287225672 |
| RP1-193H18.2 | -0.216983324 | 0.08155841 | 7.07807595 | 0.007803275 | 0.804943392 |
| LINC00675 | -0.179229541 | 0.068970064 | 6.753018301 | 0.009358923 | 0.835914001 |
| CTD-2619J13.17 | -0.226402494 | 0.093009053 | 5.925322193 | 0.014924809 | 0.797397089 |
| RP11-449D8.1 | -0.15412901 | 0.063789878 | 5.838015427 | 0.015683507 | 0.857161432 |
| AF064858.6 | -0.154490905 | 0.064198556 | 5.791023446 | 0.016108209 | 0.856851285 |
| RP11-150O12.3 | -0.138088527 | 0.058861874 | 5.503602026 | 0.018977344 | 0.87102158 |
| TP53TG1 | -0.234242224 | 0.100625757 | 5.418911327 | 0.019919777 | 0.791170152 |
| LINC00959 | -0.248083333 | 0.111140327 | 4.982551937 | 0.025604188 | 0.780294916 |
| SUCLG2-AS1 | -0.189452248 | 0.087883678 | 4.647111305 | 0.031105851 | 0.827412227 |
| LINC01315 | -0.179716406 | 0.085602037 | 4.407651488 | 0.035778065 | 0.835507123 |
| RP11-474D1.3 | -0.066106315 | 0.031730698 | 4.340368796 | 0.037218861 | 0.936031345 |
| MAFTRR | -0.183606338 | 0.088221148 | 4.33141629 | 0.037415101 | 0.83226337 |
| KBTBD11-OT1 | -0.171715939 | 0.084049421 | 4.173993502 | 0.041048937 | 0.84221838 |
| LINC01207 | -0.120084377 | 0.05931491 | 4.098691832 | 0.042916407 | 0.886845604 |
| LINC01132 | -0.213772051 | 0.106162655 | 4.054696037 | 0.044048648 | 0.80753244 |
| LINC00920 | -0.10853638 | 0.055495914 | 3.824975453 | 0.050494088 | 0.897146256 |
| RP11-805I24.3 | -0.129970029 | 0.067746141 | 3.680588884 | 0.055049404 | 0.878121749 |
| RP11-430C7.5 | 0.14836072 | 0.077655581 | 3.649997068 | 0.05606931 | 1.159931232 |
| RP11-57A19.2 | 0.120665828 | 0.063559637 | 3.604174214 | 0.057634686 | 1.128247821 |
| LINC01234 | 0.061976704 | 0.033220985 | 3.48042232 | 0.06209889 | 1.063937559 |
| LINC00941 | 0.074740905 | 0.04150572 | 3.24265539 | 0.071744271 | 1.077604913 |
| LINC00941 | 0.074740905 | 0.04150572 | 3.24265539 | 0.071744271 | 1.077604913 |
| RP11-429J17.7 | 0.154468331 | 0.08761907 | 3.108004459 | 0.077908361 | 1.167037319 |
| RP11-57A1.1 | -0.148761423 | 0.085165455 | 3.051080724 | 0.080683437 | 0.86177469 |
| LINC00961 | -0.180742825 | 0.103649903 | 3.040775336 | 0.081197127 | 0.834649982 |
| RP11-367J11.3 | 0.170616182 | 0.097980185 | 3.032242521 | 0.081625129 | 1.18603544 |
| RP11-383I23.2 | 0.138164299 | 0.079545733 | 3.016879064 | 0.082401904 | 1.148164177 |
| FENDRR | -0.097123947 | 0.056389942 | 2.966534114 | 0.085003792 | 0.907443524 |
| C9orf163 | 0.155251298 | 0.091785058 | 2.861056991 | 0.09074785 | 1.167951428 |
| AC021218.2 | -0.106004244 | 0.062710493 | 2.857367309 | 0.090956254 | 0.899420831 |
| AC021218.2 | -0.106004244 | 0.062710493 | 2.857367309 | 0.090956254 | 0.899420831 |
| CASC21 | -0.07226693 | 0.043394236 | 2.773416717 | 0.095841386 | 0.930282542 |
| RP11-458F8.4 | 0.199286471 | 0.120046374 | 2.75586245 | 0.096898551 | 1.220531563 |
| PSMG3-AS1 | -0.188267767 | 0.11501731 | 2.679325855 | 0.101658755 | 0.828392861 |
| RP11-211G23.2 | 0.099242082 | 0.06095802 | 2.65051352 | 0.103516451 | 1.104333606 |
| MIR3142HG | -0.111496132 | 0.068539528 | 2.646289684 | 0.103791895 | 0.894494851 |
| LINC00324 | -0.164928963 | 0.101698722 | 2.630043133 | 0.104858865 | 0.847953938 |
| RP1-122P22.4 | 0.070979985 | 0.044790585 | 2.511298493 | 0.113032762 | 1.073559738 |
| CYP4A22-AS1 | 0.157354591 | 0.10320287 | 2.324744833 | 0.127331099 | 1.170410557 |
| ELFN1-AS1 | 0.106708744 | 0.070268871 | 2.306078358 | 0.128868817 | 1.112610153 |
| LINC01473 | -0.131646575 | 0.087269686 | 2.275581007 | 0.131425864 | 0.876650771 |
| RP11-33O4.1 | -0.190386853 | 0.126906489 | 2.250641145 | 0.133559056 | 0.826639284 |
| C2orf48 | 0.113472963 | 0.076219339 | 2.216432143 | 0.136548296 | 1.120161603 |
| RP11-426C22.4 | 0.077691905 | 0.053256246 | 2.128190249 | 0.144611405 | 1.080789622 |
| U91328.19 | 0.208965877 | 0.143895713 | 2.108895383 | 0.146444994 | 1.232402944 |
| RP4-564F22.5 | 0.101414793 | 0.070819527 | 2.050673721 | 0.152138853 | 1.106735612 |
| RP11-528A4.2 | -0.134849043 | 0.095978298 | 1.974011376 | 0.16002272 | 0.873847815 |
| RP11-394O4.5 | 0.093104304 | 0.066306357 | 1.971647061 | 0.160273144 | 1.097576211 |
| AC123023.1 | -0.087394882 | 0.063410189 | 1.899563881 | 0.168127143 | 0.916315187 |
| RP11-353N14.2 | 0.095879814 | 0.070158368 | 1.867649671 | 0.171744509 | 1.100626777 |
| RP11-776H12.1 | 0.075738691 | 0.055420775 | 1.867628096 | 0.171746984 | 1.078680668 |
| AC009404.2 | 0.165068625 | 0.123086139 | 1.79850135 | 0.17989378 | 1.179474057 |
| RP11-834C11.4 | 0.102711827 | 0.07819922 | 1.725186583 | 0.189026785 | 1.108172018 |
| AL133493.2 | 0.060795342 | 0.04634105 | 1.721110977 | 0.189550101 | 1.062681406 |
| FIRRE | 0.061292142 | 0.047370956 | 1.674115023 | 0.195708266 | 1.063209477 |
| RP11-456K23.1 | 0.106775999 | 0.083233082 | 1.645717702 | 0.199542778 | 1.112684984 |
| LINC01550 | -0.072649376 | 0.058328727 | 1.551310654 | 0.212942057 | 0.929926828 |
| RP4-669P10.20 | 0.126645482 | 0.10296372 | 1.512902538 | 0.218696718 | 1.135014563 |
| XXbac-BPG181B23.7 | -0.11927373 | 0.097674072 | 1.49118324 | 0.222032964 | 0.887564814 |
| FLJ22763 | -0.062366028 | 0.051217038 | 1.482747676 | 0.223345117 | 0.939538926 |
| CASC15 | -0.089034692 | 0.073147415 | 1.481564478 | 0.223529905 | 0.914813837 |
| PTGES2-AS1 | 0.103244198 | 0.085528085 | 1.457182075 | 0.227379015 | 1.108762134 |
| AC007405.6 | -0.08071311 | 0.067468934 | 1.431134543 | 0.23157937 | 0.922458297 |
| CTD-3157E16.2 | -0.075311731 | 0.063398787 | 1.411118099 | 0.234870871 | 0.927454325 |
| CTD-2357A8.3 | 0.072684817 | 0.06140139 | 1.401299596 | 0.236506097 | 1.075391539 |
| RP11-1008C21.1 | -0.06772874 | 0.057510006 | 1.38694476 | 0.238921735 | 0.934513935 |
| AF064858.8 | -0.068169361 | 0.058516606 | 1.357126047 | 0.244036184 | 0.93410226 |
| RP11-326C3.12 | -0.072895819 | 0.063028385 | 1.33762042 | 0.247454048 | 0.929697682 |
| RP11-309L24.4 | 0.11087071 | 0.096861714 | 1.31017517 | 0.252362942 | 1.117250449 |
| MIR9-3HG | 0.079573396 | 0.06956215 | 1.308548399 | 0.252657644 | 1.082825032 |
| LINC01091 | -0.096495228 | 0.08551412 | 1.273315474 | 0.25914562 | 0.90801423 |
| CTA-126B4.7 | -0.065413125 | 0.05863439 | 1.244586194 | 0.26458901 | 0.936680417 |
| AC104088.1 | 0.093814757 | 0.084854651 | 1.222337165 | 0.26890247 | 1.098356264 |
| RP11-473M20.9 | -0.053256732 | 0.048235722 | 1.219021786 | 0.269552712 | 0.948136564 |
| CTB-193M12.5 | -0.166557 | 0.154497909 | 1.162199188 | 0.281009915 | 0.846574561 |
| CTD-3157E16.1 | -0.06151056 | 0.058451005 | 1.107427754 | 0.292641782 | 0.940343016 |
| AC092580.4 | -0.061150494 | 0.058908441 | 1.077568482 | 0.299242141 | 0.940681663 |
| RP11-844P9.2 | -0.060572469 | 0.058515075 | 1.071556366 | 0.300594164 | 0.941225556 |
| LINC01146 | -0.058778276 | 0.058316229 | 1.015909029 | 0.313491368 | 0.942915813 |
| LINC01356 | 0.077979698 | 0.081357063 | 0.918697587 | 0.337817176 | 1.08110071 |
| RP11-598F7.6 | -0.071810219 | 0.075154462 | 0.91298359 | 0.339324022 | 0.930707509 |
| TINCR | 0.058099714 | 0.061437952 | 0.894282081 | 0.344319537 | 1.05982067 |
| LINC00896 | 0.069771777 | 0.075307248 | 0.858392702 | 0.354188874 | 1.072263438 |
| MIR503HG | 0.058330665 | 0.064794756 | 0.810427449 | 0.367993905 | 1.060065465 |
| AP000439.3 | -0.061228615 | 0.068425334 | 0.800709567 | 0.370881305 | 0.940608178 |
| LINC00877 | -0.06793908 | 0.076517374 | 0.788350233 | 0.374599445 | 0.934317391 |
| LINC01183 | -0.087055651 | 0.098180195 | 0.78622375 | 0.375244429 | 0.916626083 |
| AF064858.11 | 0.03814135 | 0.044058758 | 0.749424017 | 0.386658652 | 1.038878068 |
| LINC01605 | -0.06813226 | 0.078820402 | 0.747185273 | 0.387368856 | 0.934136916 |
| LINC01605 | -0.06813226 | 0.078820402 | 0.747185273 | 0.387368856 | 0.934136916 |
| SPATA3-AS1 | -0.066832703 | 0.079968958 | 0.698449779 | 0.403305082 | 0.935351669 |
| RP11-462G2.1 | -0.025150851 | 0.030104335 | 0.697986954 | 0.403460935 | 0.975162796 |
| RP11-796E10.1 | -0.070098016 | 0.086352438 | 0.658964847 | 0.416925629 | 0.932302435 |
| KIAA0125 | -0.03488233 | 0.043940418 | 0.630206111 | 0.427279721 | 0.965719046 |
| RP13-616I3.1 | -0.065584504 | 0.083322487 | 0.619552374 | 0.431213675 | 0.936519904 |
| RP11-290F5.1 | -0.051891009 | 0.066421935 | 0.61032503 | 0.434665457 | 0.949432341 |
| RP11-284N8.3 | -0.040504589 | 0.052171993 | 0.602744972 | 0.437532607 | 0.960304758 |
| BLACAT1 | 0.05064996 | 0.067372081 | 0.565194965 | 0.452174652 | 1.051954602 |
| KB-68A7.1 | 0.058605164 | 0.078652317 | 0.55519906 | 0.456201108 | 1.060356491 |
| RP11-325F22.2 | -0.052799604 | 0.071621636 | 0.543466613 | 0.46099959 | 0.948570083 |
| RP11-54H7.4 | 0.026854789 | 0.036870153 | 0.530510037 | 0.466392508 | 1.027218629 |
| RP11-416I2.1 | -0.044459519 | 0.061808467 | 0.517408515 | 0.471949234 | 0.95651432 |
| RP11-706O15.1 | -0.103700375 | 0.146471654 | 0.501248952 | 0.478951857 | 0.901495368 |
| RP11-1143G9.5 | 0.037770024 | 0.05349272 | 0.498545912 | 0.480139733 | 1.038492377 |
| CTD-3252C9.4 | 0.074852423 | 0.10650605 | 0.493927465 | 0.482180564 | 1.077725092 |
| ZNF582-AS1 | 0.059357653 | 0.084976673 | 0.487926035 | 0.484853914 | 1.061154698 |
| RP11-390F4.3 | 0.049706433 | 0.071402407 | 0.484617944 | 0.48633799 | 1.050962523 |
| RP11-349K16.1 | -0.041791046 | 0.0601064 | 0.483420499 | 0.486877043 | 0.959070161 |
| RP11-629O1.2 | 0.053395679 | 0.077121923 | 0.479354185 | 0.48871499 | 1.054846943 |
| LINC00702 | 0.038152346 | 0.055264759 | 0.476591119 | 0.489970471 | 1.038889492 |
| RP11-128M1.1 | 0.045302694 | 0.066013655 | 0.470956175 | 0.492547612 | 1.046344534 |
| RP11-1029J19.5 | -0.054237996 | 0.079930183 | 0.460453379 | 0.497412162 | 0.947206648 |
| RP11-158I9.8 | 0.078771119 | 0.117892521 | 0.446438379 | 0.504031182 | 1.081956654 |
| AC016735.2 | -0.020813351 | 0.031446578 | 0.43806369 | 0.508058513 | 0.979401752 |
| AC007405.4 | 0.05257482 | 0.080427086 | 0.427317739 | 0.513308037 | 1.053981418 |
| AP001610.9 | -0.050755563 | 0.077745032 | 0.426208403 | 0.513855316 | 0.950510982 |
| RP11-490M8.1 | -0.068487892 | 0.105566409 | 0.420897262 | 0.516489667 | 0.933804767 |
| LINC01124 | -0.03957397 | 0.061664563 | 0.41185839 | 0.521027764 | 0.961198852 |
| ZNF667-AS1 | 0.049484439 | 0.077261485 | 0.410214685 | 0.52186056 | 1.050729242 |
| LINC00570 | 0.043438435 | 0.068404957 | 0.4032489 | 0.525416142 | 1.044395694 |
| RP11-1029J19.4 | -0.044762905 | 0.070576522 | 0.402268498 | 0.52592003 | 0.956224171 |
| RP11-141M1.3 | -0.051906944 | 0.082848052 | 0.392542168 | 0.530966148 | 0.949417212 |
| CRNDE | 0.040998377 | 0.065896899 | 0.387082357 | 0.533837039 | 1.041850415 |
| CTC-490G23.2 | 0.028849692 | 0.046838822 | 0.37937651 | 0.537937107 | 1.029269875 |
| RP11-351J23.1 | -0.042007848 | 0.06950553 | 0.36527685 | 0.545590113 | 0.958862255 |
| AC073283.4 | 0.055811392 | 0.092366606 | 0.36510328 | 0.545685575 | 1.057398231 |
| RP11-435O5.2 | 0.088662744 | 0.148897359 | 0.354575189 | 0.551534583 | 1.09271207 |
| LINC01579 | 0.035805837 | 0.061617547 | 0.337674944 | 0.561174372 | 1.036454585 |
| RP11-465B22.8 | 0.053704178 | 0.092646984 | 0.33601106 | 0.562140813 | 1.055172413 |
| LINC01484 | 0.045545809 | 0.080172579 | 0.322734302 | 0.569969045 | 1.046598947 |
| FAM83H-AS1 | 0.06570274 | 0.117745122 | 0.311373119 | 0.576838805 | 1.067909223 |
| RP5-1057J7.7 | -0.041333428 | 0.074636971 | 0.306686629 | 0.579720555 | 0.959509149 |
| RP11-175K6.1 | 0.044070609 | 0.080453003 | 0.300063782 | 0.583842438 | 1.045056142 |
| RP11-440I14.3 | -0.035130805 | 0.064815137 | 0.293780733 | 0.587807872 | 0.965479119 |
| RP11-102N12.3 | 0.043130035 | 0.080985838 | 0.283623009 | 0.594336341 | 1.044073652 |
| LINC00996 | -0.035022516 | 0.066001331 | 0.281571889 | 0.595672791 | 0.965583675 |
| SNHG15 | 0.090670124 | 0.171210237 | 0.280458729 | 0.596400707 | 1.094907762 |
| CCAT1 | -0.040397499 | 0.077393894 | 0.272455486 | 0.601689348 | 0.960407602 |
| CTD-2227E11.1 | 0.040975012 | 0.079661236 | 0.264572133 | 0.606996295 | 1.041826072 |
| LINC01215 | -0.033068588 | 0.065479568 | 0.255046684 | 0.613543757 | 0.967472201 |
| RP5-1158E12.3 | -0.041443255 | 0.083112266 | 0.248643709 | 0.618031707 | 0.959403775 |
| RP11-22L13.1 | -0.030595689 | 0.061449465 | 0.247904414 | 0.618554525 | 0.969867622 |
| RP11-353N14.4 | -0.030046147 | 0.060607778 | 0.245765464 | 0.620072655 | 0.970400751 |
| LINC01268 | -0.048235849 | 0.097480486 | 0.244852464 | 0.620723168 | 0.952909018 |
| LINC00152 | 0.05459529 | 0.114187183 | 0.22859969 | 0.632564087 | 1.056113109 |
| LINC01140 | 0.033778115 | 0.072152396 | 0.219164036 | 0.63967762 | 1.034355073 |
| CASC19 | -0.032564577 | 0.069827938 | 0.217486565 | 0.64096174 | 0.96795994 |
| LINC01082 | -0.031425103 | 0.067960783 | 0.213814261 | 0.643794111 | 0.969063534 |
| RP11-400K9.4 | -0.026188759 | 0.057349428 | 0.208531495 | 0.647920846 | 0.974151192 |
| AC144831.1 | 0.034465834 | 0.075903907 | 0.206181323 | 0.649777052 | 1.035066664 |
| RP11-436K8.1 | -0.025327795 | 0.056535805 | 0.200700103 | 0.654156336 | 0.974990263 |
| RP11-159D12.2 | -0.039335954 | 0.089134054 | 0.194756541 | 0.658986927 | 0.96142766 |
| RP1-278O22.1 | -0.022300571 | 0.052038677 | 0.183645156 | 0.668259186 | 0.977946248 |
| AC018359.1 | -0.027649327 | 0.064539316 | 0.183535639 | 0.668352212 | 0.972729417 |
| RP11-532F6.3 | 0.034201484 | 0.081702872 | 0.175232739 | 0.675502571 | 1.03479308 |
| RP5-884M6.1 | 0.023398304 | 0.056903544 | 0.169079177 | 0.680931472 | 1.023674192 |
| AL163953.2 | 0.031903219 | 0.078611444 | 0.164701468 | 0.684864364 | 1.032417582 |
| RP11-160O5.1 | -0.041753452 | 0.104192828 | 0.160586534 | 0.688617082 | 0.959106217 |
| RP11-253E3.3 | -0.04604144 | 0.115133621 | 0.159916573 | 0.689233338 | 0.955002386 |
| AC006129.2 | -0.028761614 | 0.072016513 | 0.159500603 | 0.689616717 | 0.971648064 |
| LINC01559 | 0.026221585 | 0.066141817 | 0.157168554 | 0.691776852 | 1.026568396 |
| EPB41L4A-AS2 | -0.028781659 | 0.072993412 | 0.155476339 | 0.69335596 | 0.971628588 |
| RP1-140K8.5 | 0.020869951 | 0.054508746 | 0.146592076 | 0.701813601 | 1.021089252 |
| RP1-140K8.5 | 0.020869951 | 0.054508746 | 0.146592076 | 0.701813601 | 1.021089252 |
| RP11-401P9.4 | 0.020188795 | 0.052979814 | 0.145211146 | 0.703154422 | 1.020393968 |
| RP11-47L3.1 | 0.027343743 | 0.074193187 | 0.135827548 | 0.712464742 | 1.027721014 |
| AC092614.2 | 0.037732697 | 0.105348288 | 0.128286431 | 0.720215384 | 1.038453614 |
| CTA-217C2.2 | 0.029873005 | 0.083583291 | 0.12773764 | 0.720789361 | 1.03032368 |
| LINCR-0001 | -0.020112229 | 0.056349303 | 0.127392336 | 0.721151225 | 0.980088673 |
| RP11-43F13.4 | -0.02506182 | 0.07075673 | 0.125455496 | 0.723191272 | 0.975249621 |
| MIR4435-2HG | 0.05127403 | 0.148472751 | 0.119261808 | 0.729836501 | 1.052611301 |
| RP11-774O3.3 | 0.03163873 | 0.092292475 | 0.117518297 | 0.73174184 | 1.032144555 |
| C5orf66-AS1 | -0.024749778 | 0.075701221 | 0.106889949 | 0.743713052 | 0.975553986 |
| LINC01588 | 0.041563559 | 0.130386541 | 0.101615491 | 0.749899527 | 1.042439416 |
| RP11-863P13.3 | 0.018769817 | 0.059799395 | 0.09852048 | 0.753612392 | 1.018947078 |
| RP11-94C24.13 | 0.026494683 | 0.088609415 | 0.08940416 | 0.764936014 | 1.026848788 |
| LINC01572 | -0.032372034 | 0.10993286 | 0.086713142 | 0.768398057 | 0.968146332 |
| LINC00704 | -0.018313853 | 0.063977364 | 0.081942036 | 0.774682672 | 0.981852827 |
| LINC01133 | 0.02188583 | 0.078890423 | 0.076962202 | 0.781457149 | 1.022127082 |
| LINC01197 | -0.019768215 | 0.071333061 | 0.076798578 | 0.781683693 | 0.980425895 |
| RP4-794I6.4 | 0.016564712 | 0.063875163 | 0.067251772 | 0.795381046 | 1.016702668 |
| PVT1 | 0.032321932 | 0.135973318 | 0.056505047 | 0.812107654 | 1.03284996 |
| LINC01186 | 0.021200609 | 0.092722806 | 0.052278551 | 0.81914482 | 1.021426939 |
| MIR22HG | -0.020364654 | 0.101896426 | 0.039942585 | 0.841592882 | 0.979841305 |
| RP11-37B2.1 | 0.020301265 | 0.109516436 | 0.034362726 | 0.852937379 | 1.020508738 |
| AC003090.1 | -0.012353634 | 0.066971506 | 0.034025875 | 0.85365179 | 0.987722359 |
| LINC00294 | 0.033735458 | 0.186217969 | 0.032819356 | 0.856241101 | 1.034310952 |
| TUSC8 | 0.008867048 | 0.051329604 | 0.029841614 | 0.862850098 | 1.008906477 |
| RP3-522D1.1 | 0.013334148 | 0.077256696 | 0.029789161 | 0.862969494 | 1.013423444 |
| RP11-527N22.2 | 0.012795484 | 0.076113512 | 0.028261157 | 0.86649631 | 1.012877697 |
| LINC00346 | 0.012967807 | 0.082021927 | 0.024996153 | 0.874376647 | 1.013052254 |
| LINC01272 | 0.011138078 | 0.081735551 | 0.018569429 | 0.891608083 | 1.011200338 |
| MNX1-AS1 | 0.014801286 | 0.111791443 | 0.017529995 | 0.894667212 | 1.014911368 |
| AC144831.3 | 0.010360644 | 0.078778629 | 0.017296439 | 0.895367189 | 1.010414502 |
| RP11-1055B8.9 | -0.007469164 | 0.057265589 | 0.017012044 | 0.896226053 | 0.992558661 |
| RP11-546K22.1 | -0.008115567 | 0.066067535 | 0.015089038 | 0.902235822 | 0.991917275 |
| RP5-1009E24.8 | 0.008455959 | 0.069788534 | 0.014681067 | 0.903559988 | 1.008491812 |
| RP11-502I4.3 | -0.013299256 | 0.125217108 | 0.011280475 | 0.91541607 | 0.986788788 |
| LINC00460 | -0.004595067 | 0.045411133 | 0.010239037 | 0.919401123 | 0.995415474 |
| LINC01615 | 0.006731584 | 0.066695726 | 0.010186817 | 0.919606216 | 1.006754292 |
| C14orf132 | -0.006305066 | 0.065555118 | 0.009250518 | 0.923377923 | 0.993714769 |
| RP11-93H24.3 | 0.009115131 | 0.098214326 | 0.008613431 | 0.926055642 | 1.009156801 |
| LINC00863 | 0.011661627 | 0.128474708 | 0.008239161 | 0.927675486 | 1.011729889 |
| RP11-10A14.4 | -0.007070534 | 0.078144346 | 0.008186707 | 0.927905448 | 0.992954404 |
| AC058791.1 | 0.007342754 | 0.081840525 | 0.008049727 | 0.928509507 | 1.007369778 |
| RP11-734K21.5 | 0.004928008 | 0.061493756 | 0.006422155 | 0.936127223 | 1.004940171 |
| RP11-353N14.5 | 0.005211017 | 0.066353208 | 0.006167671 | 0.937402871 | 1.005224618 |
| FGF14-AS2 | -0.005177576 | 0.07118002 | 0.005290988 | 0.942013674 | 0.994835805 |
| LINC01348 | 0.003780122 | 0.068824358 | 0.003016666 | 0.956198863 | 1.003787276 |
| LINC00865 | 0.002296397 | 0.064784793 | 0.001256458 | 0.971723667 | 1.002299036 |
| RP11-620J15.3 | 0.003389633 | 0.095995281 | 0.001246825 | 0.97183222 | 1.003395384 |
| AC009014.3 | 0.001580316 | 0.04665243 | 0.001147465 | 0.972977421 | 1.001581566 |
| RP11-138J23.1 | 0.001485109 | 0.076267556 | 0.000379173 | 0.984464287 | 1.001486213 |
| LINC01273 | 0.001364375 | 0.077645967 | 0.000308766 | 0.985980501 | 1.001365306 |
| LINC00092 | 0.001232931 | 0.087383685 | 0.000199075 | 0.988742703 | 1.001233691 |
| RP11-321G12.1 | 0.0007459 | 0.053382384 | 0.000195238 | 0.988851707 | 1.000746178 |
| AC016735.1 | -0.00104845 | 0.079468812 | 0.000174061 | 0.989473635 | 0.998952099 |
| LINC01270 | -0.000696666 | 0.083932128 | 6.88959E-05 | 0.993377352 | 0.999303576 |
| PCAT1 | -0.000386078 | 0.091218069 | 1.79138E-05 | 0.996622985 | 0.999613996 |
